# The effects of ACE2 expression mediating pharmacotherapy in COVID-19 patients

**DOI:** 10.1007/s12471-021-01573-8

**Published:** 2021-04-16

**Authors:** R. R. J. van Kimmenade, E. Belfroid, J. Hoogervorst-Schilp, H. J. Siebelink, C. W. Janssen, Y. Pinto

**Affiliations:** 1 Department of Cardiology, Radboud UMC, Nijmegen, The Netherlands; 2 Knowledge Institute of Medical Specialists, Utrecht, The Netherlands; 3 Department of Cardiology, Leiden University Medical Center, Leiden, The Netherlands; 4 Netherlands Society of Cardiology, Utrecht, The Netherlands; 5 Department of Experimental Cardiology, Amsterdam University Medical Center, Amsterdam, The Netherlands

**Keywords:** COVID-19, ACE2, RAAS, Mortality

## Abstract

**Background:**

There has been debate on the use of angiotensin-converting enzyme‑2 (ACE2) expression mediating pharmacotherapy in COVID-19 infected patients. Although it has been suggested that these drugs might lead to a higher susceptibility and severity of COVID-19 infection, experimental data suggest these agents may reduce acute lung injury via blocking angiotensin-II-mediated pulmonary permeability, inflammation and fibrosis.

**Methods:**

A systematic literature search was performed to answer the question: What is the effect of medications that influence ACE2 expression (ACE inhibitors (ACEIs), angiotensin receptor blockers (ARBs), nonsteroidal anti-inflammatory drugs (NSAIDs) and thiazolidinediones) on the outcomes of COVID-19? Relevant outcome measures were mortality (crucial), hospital admission, length of stay, thromboembolic complications (pulmonary embolism, stroke, transient ischaemic attack), need for mechanical ventilation, acute kidney injury and use of renal replacement therapy. Medline and Embase databases were searched with relevant search terms until 24 June 2020. After systematic analysis, nine studies were included.

**Results:**

The results were described for two different groups, an overall group in which all users were compared with non-users and a group in which only hypertensive patients were included. Within each group a distinction was made between results for ACEI/ARB use, ACEI use, ARB use, NSAID use and thiazolidinedione use. None of the studies demonstrated increased mortality in the two groups. Furthermore, none of the studies showed an effect on other outcome measures in COVID-19, such as ICU admission, length of hospital stay, thromboembolic complications, need for mechanical ventilation, acute kidney failure or need for renal replacement therapy. However, the level of evidence of all studies varied from ‘moderate’ to ‘very low’, according to the GRADE methodology.

**Conclusion:**

Analysis of the literature demonstrated that there was insufficient evidence to answer our objective on the effect of ACE2 expression mediating pharmacotherapy on outcome in COVID-19 patients, especially due to the low scientific quality of the described studies. Randomised controlled studies are needed to answer this question.

**Supplementary Information:**

The online version of this article (10.1007/s12471-021-01573-8) contains supplementary material, which is available to authorized users.

## Clinical question

*What is the effect of medications that influence ACE2 expression (ACEIs, ARBs, NSAIDs and thiazolidinediones) on the outcomes of COVID-19 patients?*

## Introduction

COVID-19 uses the angiotensin-converting enzyme‑2 (ACE2) to infect the cell. ACE2 degrades angiotensin II. Angiotensin II increases blood pressure. Pharmaceutical agents given for high blood pressure such as ACE inhibitors (ACEIs) or angiotensin receptor blockers (ARBs) can increase the level of ACE2. It was initially thought that these agents would make ACE2 more sensitive to COVID-19 as it is, after all, the ‘gateway’ to the virus. However, ACE2 also appears to protect against high levels of angiotensin II; increased activity of angiotensin II is common in the lung in COVID-19-induced pneumonia. It is therefore unclear whether drugs that increase or decrease ACE2 can be harmful.

## Methods

A review of the literature was performed to answer the following question: What is the effect of using medications that influence ACE2 expression (ACEIs, ARBs, NSAIDs and thiazolidinediones) on the outcomes in patients with COVID-19? This question was structured in a PICO format.

**P**opulation: All proven COVID-19 patients
**I**ntervention: Using medications before and during COVID-19 that influence ACE2 expression: ACEIs, ARBs, NSAIDs and thiazolidinediones
**C**omparison: No use of medications that influence ACE2 expression before and during COVID-19
**O**utcome: Mortality, ICU admission, hospital admission, length of stay, thromboembolic complications (pulmonary embolism, stroke, transient ischaemic attack), ventilation

### Relevant outcome measures

Mortality was considered to be a critical outcome measure for decision-making and the other outcomes important for decision-making. A priori, the working group did not define minimal clinically relevant differences for the outcome measures.

### Search and select

The databases Medline (via Ovid) and Embase (via Embase.com) were searched with relevant search terms until 24 June 2020. The systematic literature search resulted in 567 hits (see Table S1 of the Electronic Supplementary Material for details). A total of 64 studies were initially selected based on the title and abstract screening. After reading the full text, 56 studies were excluded (see Table S2 of the Electronic Supplementary Material for the reasons for exclusion). Nine studies were included in the analysis of the literature. Important study characteristics and results are summarised in the evidence tables (Tables S3, S4, S5 of the Electronic Supplementary Material). The assessment of the risk of bias is summarised in Table S6 of the Electronic Supplementary Material.

### Description of studies

**Zhang** [1] assessed the relationship between ACEI/ARB use and COVID-19 infection in a systematic review. A comprehensive search of the PubMed, Embase and Cochrane Library databases was performed to identify all relevant articles published between 1 January 2020 and 9 May 2020. Observational studies that met all the following criteria were included: (1) Study design: case-control, case-crossover, self-controlled case series or cohort study; (2) Antihypertensive treatment: ACEI/ARB use versus non-ACEI/ARB use; (3) Outcomes: the incidence of COVID-19, critical cases or death; (4) Adequate data were used to extract the risk estimates if the adjusted data were not provided in the publication. Editorials, correspondence, conference abstracts and commentary articles were excluded. Twelve articles (case-control and cohort studies) involving more than 19,000 COVID-19 cases were included. Information was not given for the duration of follow-up and the number of patients for whom complete outcome data were not available.

**Mackey** [2] evaluated whether the use of ACEIs or ARBs either increased the risk for severe acute respiratory syndrome coronavirus 2 (SARS-CoV-2) infection or was associated with worse COVID-19 disease outcomes, and the efficacy of these medications for COVID-19 treatment in a systematic review. Medline (OVID) and the Cochrane Database of Systematic Reviews were searched from 2003 to 4 May 2020, with planned ongoing surveillance for one year; the World Health Organisation database of COVID-19 publications and medRxiv.org through to 17 April 2020; and ClinicalTrials.gov to 24 April 2020, with planned ongoing surveillance. Observational studies and trials in adults that examined associations and effects of ACEIs or ARBs on the risk of SARS-CoV‑2 infection and COVID-19 disease severity and mortality were included. Nineteen studies were included. Some of the included studies describe a composite outcome measure ‘severe COVID-19’.

**Felice** [3] investigated the association between chronic use of ACEIs or ARBs and COVID-19-related outcomes in hypertensive patients. A single-centre study was conducted on 133 consecutive hypertensive subjects presenting to the emergency department with acute respiratory symptoms and/or fever, who were diagnosed with COVID-19 infection between 9 and 31 March 2020. All patients were grouped according to their chronic antihypertensive medications (ACEIs, *n* = 40; ARBs, *n* = 42; not on renin-angiotensin-aldosterone system (RAAS) inhibitors, *n* = 51).

**Gao** [4] investigated whether treatment of hypertension, especially with RAAS inhibitors, had an impact on the mortality of patients with COVID-19. Consecutive patients admitted to Huo Shen Shan Hospital in Wuhan, China (solely for the treatment of COVID-19) from 5 February to 15 March 2020 were included. In total, 2877 consecutive hospitalised patients with confirmed COVID-19 were enrolled in the study. The median time from symptom onset to discharge (last follow-up) was 39 (30–50) days. There were 710/850 (83.5%) patients with hypertension taking antihypertensive medications. A total of 183 (25.7%) patients were treated with RAAS inhibitors and 527 (74.2%) treated with beta-blockers, calcium channel blockers or diuretics (non-RAAS inhibitors). For the outcome measures of interest, the group of 710 patients was used, meaning that hypertensive patients treated with RAAS inhibitors were compared with hypertensive patients taking antihypertensive medications other than RAAS inhibitors. The medical history and blood pressure at admission did not differ significantly between the RAAS inhibitor-treated [RAASi (+)] and non-RAAS inhibitor-treated [RAASi (–)]patients. Fourteen patients reported shivering at admission in the RAASi (–) cohort, compared with none in the RAASi (+) cohort.

**Jung** [5] aimed to assess the associations between prior use of RAAS inhibitors and clinical outcomes among Korean patients with COVID-19. Among 5179 confirmed COVID-19 cases, 762 patients were RAAS inhibitor users and 4417 patients were non-users. Relative to non-users, RAAS inhibitor users were more likely to be older, male and have comorbidities. Among 1954 hospitalised patients with COVID-19, 377 patients were on RAAS inhibitors and 1577 patients were non-users.

**López-Otero** [6] performed a single-centre, retrospective, observational cohort study on 965 patients diagnosed with COVID-19 from 10 March to 6 April 2020. In total, 210 patients were under ACEI or ARB treatment at the time of diagnosis; 165 (78.57%) had been taking these medications for more than 1 year. During the study period, 38 patients died (3.94%), 35 (3.6%) of whom had heart failure. The cohort of patients receiving ACEI/ARB treatment was older (72.1 ± 13.2 vs 56.0 ± 20.5, *p* < 0.01) and had more cardiovascular risk factors (hypertension, diabetes, smoking and dyslipidaemia) and cardiovascular comorbidities (coronary artery diseases and ventricular dysfunction) than the cohort without ACEIs/ARBs. There were fewer women in the ACEI/ARB group (43.8% vs 59.5%, *p* < 0.01). Renal impairment and peripheral vasculopathy were also more prevalent in patients taking ACEIs/ARBs.

**Selçuk** [7] aimed to determine the relation between the use of ACEIs and ARBs and in-hospital mortality of hypertensive patients diagnosed with COVID-19 pneumonia. All patients were on ACEIs/ARBs or other antihypertensive therapy. In total, 113 hypertensive COVID-19 patients were included, 74 of whom were receiving ACEIs/ARBs. During in-hospital follow-up, 30.9% (*n* = 35) of the patients died.

**Imam** [8] evaluated mortality predictors of COVID-19 in a large cohort of hospitalised patients in the US. A retrospective, multicentre cohort study of inpatients diagnosed with COVID-19 by reverse transcription-polymerase chain reaction from 1 March to 1 April 2020 was performed, and outcome data were evaluated from 1 March to 17 April 2020. Measures included demographics, comorbidities, clinical presentation, laboratory values and imaging on admission. The primary outcome was mortality. Secondary outcomes included length of stay, time to death and development of acute kidney injury in the first 48 h. A total of 1305 patients were hospitalised during the evaluation period. Mean age was 61.0 ± 16.3, 53.8% were male and 66.1% were African-American. Mean body mass index (BMI) was 33.2 ± 8.8 kg/m^2^. The median Charlson Comorbidity Index was 2 (1–4), 72.6% of the patients had at least one comorbidity, with hypertension (56.2%) and diabetes mellitus (30.1%) being the most prevalent. ACEI/ARB use and NSAID use were widely prevalent (43.3% and 35.7%, respectively). Mortality occurred in 200 (15.3%) of the patients with a median time of 10 (6–14) days.

**Zhou** [9] aimed to explore the clinical characteristics of COVID-19 complicated by hypertension. A retrospective, single-centre study was conducted in which 110 discharged patients with COVID-19 at Wuhan Fourth Hospital in Wuhan, China, from 25 January to 20 February 2020, were included. All study cases were grouped according to whether they had a history of hypertension. Then, a subgroup analysis for all hypertensive patients was carried out based on whether or not they were taking ACEI or ARB medication. The mean age of these 110 patients was 57.7 years (range 25–86 years), 60 (54.5%) were males. The main underlying diseases included hypertension (36 (32.7%)) and diabetes (11 (10.0%)).

Table 1 shows the characteristics of the included studies.

## Results

The results are described for two different groups, an overall group in which all users were compared with non-users and a group in which only hypertensive patients were included. Within each group a distinction was made between the results for ACEI/ARB use, ACEI use, ARB use, NSAID use and thiazolidinedione use.

### Mortality (overall group)

#### ACEIs/ARBs

We were unable to provide a pooled estimate for mortality since some studies did not provide the absolute number of events or use a composite outcome measure. Therefore, the results of each of the studies are described separately.

**Fig. 1:**
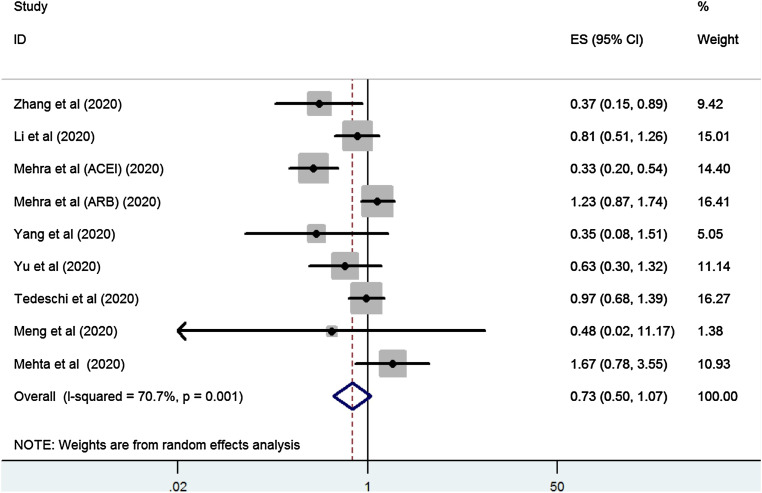
Forest plot of ACEI/ARB exposure and risk of mortality in COVID-19 patients. Reprinted from Zhang et al. [1], with permission from Elsevier

**Zhang** [1] performed a meta-analysis to study the relation between ACEI/ARB use and mortality. In this meta-analysis, all studies that assessed this relation were included, irrespective of the type of patients in the intervention (all patients using ACEIs/ARBs or only hypertensive patients) and control group (all COVID-19 patients not on ACEIs/ARBs, hypertensive patients not on ACEIs/ARBs but on other or no blood pressure lowering medication). Overall, the risk of mortality in ACEI/ARB-exposed patients was similar to non-ACEI/ARB exposed COVID-19 patients (pooled odds ratio (OR) 0.73, 95% confidence incidence (CI) 0.5–1.07, *p* = 0.11) (Fig. 1).

**Imam** [8] and **López-Otero** [6] also studied the relation between ACEI/ARB use and mortality between users of ACEIs/ARBs and non-users. In a multivariate analysis both Imam and López-Otero reported no statistically significant difference in mortality between users of ACEIs/ARBs and non-users (López-Otero: 8 out of 78 ACEI/ARB users died (OR 0.62, 95% CI 0.17–2.26, *p* = 0.486); Imam: adjusted OR 1.20, 95% CI 0.86–1.68, *p* = 0.278). López-Otero found that the absence of an impact on mortality remained in both the multivariate analysis and in the propensity score model, including in the evaluation of treatment taken for more than 1 year.

**Reynolds** [2] (included in the review by Mackey) studied the relation between ACEI or ARB use and the composite outcome ‘severe COVID-19’, defined as ICU admission, use of noninvasive or mechanical ventilation, or death. The mean difference between users and non-users of this medication was −0.1 (95% CI −3.7 to 3.5), meaning that there was no statistically significant difference between both groups. None of the studies showed a statistically significant difference between ACEI or ARB use and non-users with regard to mortality (or a composite outcome including mortality).

#### ARBs

**Reynolds** [2] (included in the review by Mackey) studied the relation between ARBs and the composite outcome ‘severe COVID-19’, defined as ICU admission, use of noninvasive or mechanical ventilation, or death. The mean difference between users and non-users of this medication was −1.4 (95% CI −6.1 to 3.3), meaning that there was no statistically significant difference between both groups.

**Mancia** [2] (included in the review by Mackey) studied the relation between ARBs and the composite outcome ‘severe COVID-19’ defined as assisted ventilation or death. The adjusted OR was 0.83 (95% CI 0.63–1.10).

**Jung** [5] studied the relation between ACEI/ARB use (*n* = 377) and mortality. Since most of the included patients were only taking ARBs, the results of this paper are included in the ARBs-only category. In a multivariate analysis, adjusted for age, sex, Charlson Comorbidity Index, immunosuppression and hospital type, Jung found no statistically significant difference in mortality between users and non-users (adjusted OR 0.88, 95% CI 0.53–1.44, *p* = 0.60).

In the study by **López-Otero** [6] 6 out of 50 ARB users died. López-Otero found no statistically significant difference in a multivariate analysis, adjusted for arterial oxygen saturation < 95%, diabetes mellitus, hypoxaemia, hypercapnia, lymphocytes, creatinine, elevated troponin, ferritin, C‑reactive protein and interleukin‑6 (OR 1.54, 95% CI 0.42–5.59).

**Mehra** [1] (included in the review by Zhang) studied the relation between ARB use and mortality and found an OR of 1.23 (95% CI 0.87–1.74).

None of the studies showed a statistically significant difference between ARB use and non-users with regard to mortality or a composite outcome including mortality.

#### ACEIs

**Reynolds** [2] (included in the review by Mackey) studied the relation between ACEIs and the composite outcome ‘severe COVID-19’, defined as ICU admission, use of noninvasive or mechanical ventilation, or death. The mean difference between users and non-users of this medication was −1.9 (95% CI −6.6 to 2.8), meaning that there was no statistically significant difference between both groups.

**Bean** [2] (included in the review by Mackey) found an adjusted OR for the composite outcome ‘mortality and transfer to critical care within 7 days of symptom onset’ of 0.29 (95% CI 0.10–0.75) for ACEI use vs non-users. This paper was not peer-reviewed.

**Mancia** [2] (included in the review by Mackey) studied the relation between ACEIs and the composite outcome ‘severe COVID-19’ defined as assisted ventilation or death. The adjusted OR was 0.91 (95% CI 0.69–1.21).

In the study by **López-Otero** [6] 2 out of 29 ACEI users died. They found no statistically significant difference in a multivariate analysis, adjusted for arterial oxygen saturation < 95%, diabetes mellitus, hypoxaemia, hypercapnia, lymphocytes, creatinine, elevated troponin, ferritin, C‑reactive protein and interleukin‑6 (OR 0.14, 95% CI 0.01–1.57).

**Mehra** [2] (included in the review by Zhang) studied the relation between ACEI use and mortality, and found an OR of 0.33 (95% CI 0.20–0.54).

Three studies showed no statistically significant difference between ACEI use and non-users with regard to mortality or a composite outcome including mortality. Two studies (one not peer reviewed and assessing mortality within 7 days of symptom onset) showed a statistically significant difference.

#### NSAIDs

For NSAID use, **Imam** [8] found that NSAID users had a statistically significantly lower risk of mortality compared with non-NSAID users in a multivariate analysis, adjusted for age, initial serum creatinine, Charlson Comorbidity Index, NSAID use, hypertension, ACEI/ARB use and chronic kidney disease (OR 0.57, 95% CI 0.40–0.82, *p* = 0.002).

#### Thiazolidinediones

**Reynolds** [2] studied the relation between thiazide diuretics and the composite outcome ‘severe COVID-19’, defined as ICU admission, use of noninvasive or mechanical ventilation, or death. The mean difference between users and non-users of this medication was −3.4 (95% CI −8.3 to 1.6).

### *Mortality (hypertensive patients*)

#### ACEIs/ARBs

We were unable to provide a pooled estimate for mortality since some studies did not provide the absolute number of events or use a composite outcome measure. Therefore, the results of each of the studies are described separately.

**Fig. 2:**
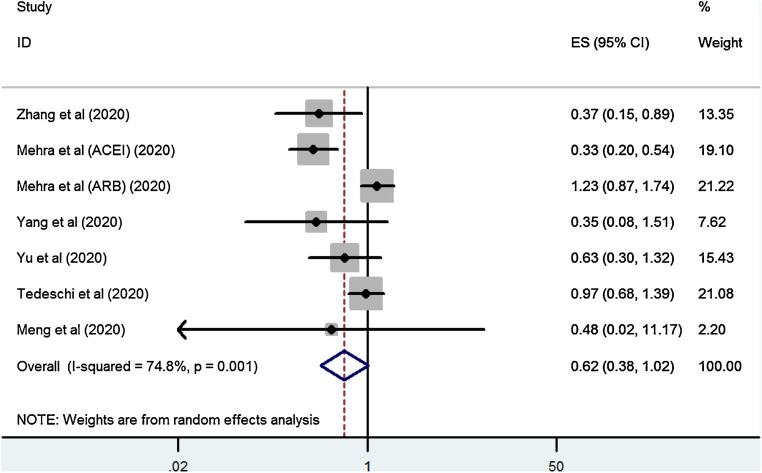
Forest plot of ACEI/ARB exposure and risk of mortality in COVID-19 patients with antihypertensive indication. Reprinted from Zhang et al. [1], with permission from Elsevier

**Zhang** [1] performed a meta-analysis of seven studies in which hypertensive ACEI/ARB users were compared with hypertensive patients on other blood pressure lowering medication or no medication. In this meta-analysis, Zhang observed no statistically significant difference in risk of mortality among those who were on ACEIs/ARBs (OR 0.62, 95% CI 0.38–1.02, *p* = 0.059, I^2^ = 74.8%) (Fig. 2).

**Zhang** [1] found in a meta-analysis of four studies that ACEI/ARB use in hypertensive patients was associated with a lower risk of mortality compared with those on non-ACEI/ARB antihypertensive drugs (OR 0.48, 95% CI 0.29–0.81, *p* = 0.006, I^2^ 0%).

**Selçuk** [7] found that ACEI/ARB use was associated with a higher risk of mortality (adjusted OR 3.66, 95% CI 1.11–18.18, *p* = 0.032). The Kaplan-Meier curve analysis showed that patients on ACEI/ARB therapy had a higher incidence of in-hospital death than those who were not (log-rank test *p* value < 0.001).

**Felice** [3] found no statistically significant association between ACEI/ARB use in hypertensive patients in a multivariate analysis (OR 0.56, 95% CI 0.17–1.83, *p* = 0.341).

**Gao** [4] found no statistically significant association between ACEI/ARB use in hypertensive patients in a multivariate analysis (adjusted OR 0.85, 95% CI 0.28–2.58, *p* = 0.774).

**Zhou** [9] found no statistically significant difference in mortality between ACEI/ARB use in hypertensive patients using Student’s unpaired t‑test. We calculated the OR, which was 0.49 (95% CI 0.082–2.966).

**Reynolds** [2] (included in the review by Mackey) studied the relation between ACEI or ARB use in hypertensive patients and the composite outcome ‘severe COVID-19’, defined as ICU admission, use of noninvasive or mechanical ventilation, or death. The mean difference between users and non-users of this medication was −0.5 (95% CI −4.3 to 3.2).

Most of the studies showed no statistically significant difference between ACEI/ARB use in hypertensive patients and non-users with regard to mortality (or a composite outcome including mortality). In the sub-analysis by Zhang [1] (including only hypertensive patients on medication other than ACEI/ARB drugs in the control group, so not including hypertensive patients on no medication) and Selçuk [7] a significant difference was found.

#### ARBs

**Reynolds** [2] (included in the review by Mackey) studied the relation between ARB use in hypertensive patients and the composite outcome ‘severe COVID-19’, defined as ICU admission, use of noninvasive or mechanical ventilation, or death. The mean difference between users and non-users of this medication was 0.1 (95% CI −4.8 to 4.9).

**Jung** [5] studied the relation between hypertensive ACEI/ARB users and hypertensive patients on other blood pressure lowering medication or no medication. Since most of the included patients were only on ARBs, the results of this paper are included in the ARBs-only category. Jung found that RAAS inhibitor use was not independently associated with a higher risk of mortality among hypertensive COVID-19 patients (adjusted OR 0.71, 95% CI 0.40–1.26, *p* = 0.25), adjusted for age, sex, Charlson Comorbidity Index, immunosuppression and hospital type.

**Mehra** [1] (included in the review by Zhang) studied the relation between ARB use and mortality, and found an OR of 1.23 (95% CI 0.87–1.74).

None of the studies showed a statistically significant difference between ARB use in hypertensive patients and non-users with regard to mortality or a composite outcome including mortality.

#### ACEIs

**Reynolds** [2] (included in the review by Mackey) studied the relation between ACEI use in hypertensive patients and the composite outcome ‘severe COVID-19’, defined as ICU admission, use of noninvasive or mechanical ventilation, or death. The mean difference between users and non-users of this medication was −3.3 (−8.2 to 1.7).

**Mehra** [1] (included in the review by Zhang) studied the relation between ACEI use and mortality, and found an OR of 0.33 (95% CI 0.20–0.54).

The study showed no statistically significant difference between ACEI use in hypertensive patients and non-users with regard to mortality (or a composite outcome including mortality) and one study found a statistically significant difference.

#### Thiazolidinediones

**Reynolds** [2] studied the relation between thiazide diuretics and the composite outcome ‘severe COVID-19’, defined as ICU admission, use of noninvasive or mechanical ventilation, or death. The mean difference between users and non-users of this medication was 0.6 (95% CI −4.5 to 5.7).

### ICU admission (overall group)

#### ACEIs/ARBs

In the review by Mackey [2] two studies assessed the relationship between ACEI/ARB use and ICU admission (Rentsch and Reynolds). Rentsch found that admission to the ICU was more likely to occur in patients taking ACEIs/ARBs compared with non-users (adjusted OR 1.69, 95% CI 1.01–2.84). This study was not peer-reviewed.

**Reynolds** [2] studied the relation between ACEI or ARB use and the composite outcome ‘severe COVID-19’, defined as ICU admission, use of noninvasive or mechanical ventilation, or death. The mean difference between users and non-users of this medication was −0.1 (95% CI −3.7 to 3.5), meaning that there was no statistically significant difference between both groups.

**López-Otero** [6] found no statistically significant difference in a multivariate analysis, adjusted for arterial oxygen saturation < 95%, diabetes mellitus, hypoxaemia, hypercapnia, lymphocytes, creatinine, elevated troponin, ferritin, C‑reactive protein and interleukin‑6 (OR 0.87, 95% CI 0.30–2.50, *p* = 0.798).

Two studies showed no statistically significant difference while one study that was not peer reviewed showed a statistically significant difference.

#### ARBs

**Reynolds** [2] (included in the review by Mackey) studied the relation between ARBs and the composite outcome ‘severe COVID-19’, defined as ICU admission, use of noninvasive or mechanical ventilation, or death. The mean difference between users and non-users of this medication was −1.4 (95% CI −6.1 to 3.3), meaning that there was no statistically significant difference between both groups.

**López-Otero** [6] found that seven ARB users were admitted to the ICU. They found no statistically significant difference in a multivariate analysis, adjusted for arterial oxygen saturation < 95%, diabetes mellitus, hypoxaemia, hypercapnia, lymphocytes, creatinine, elevated troponin, ferritin, C‑reactive protein and interleukin‑6 (OR 0.84, 95% CI 0.25–2.87 *p* = 0.786).

#### ACEIs

**Bean** [2] (included in the review by Mackey) found an adjusted OR for the composite outcome ‘mortality and transfer to critical care within 7 days of symptom onset’ of 0.29 (95% CI 0.10–0.75) for ACEI use vs non-users. This paper was not peer-reviewed.

**Reynolds** [2] (included in the review by Mackey) studied the relation between ACEI use and the composite outcome ‘severe COVID-19’, defined as ICU admission, use of noninvasive or mechanical ventilation, or death. The mean difference between users and non-users of this medication was −1.9 (95% CI −6.6 to 2.8), meaning that there was no statistically significant difference between both groups.

**López-Otero** [6] found that six ACEI users were admitted to the ICU. They found no statistically significant difference between ACEI users and non-users in a multivariate analysis, adjusted for arterial oxygen saturation < 95%, diabetes mellitus, hypoxaemia, hypercapnia, lymphocytes, creatinine, elevated troponin, ferritin, C‑reactive protein and interleukin‑6 (OR 0.97, 95% CI 0.22–4.16, *p* = 0.962).

One study showed no statistically significant difference and one study that was not peer reviewed showed a statistically significant difference.

#### Thiazolidinediones

**Reynolds** [2] studied the relation between thiazide diuretics and the composite outcome ‘severe COVID-19’, defined as ICU admission, use of noninvasive or mechanical ventilation, or death. The mean difference between users and non-users of this medication was −3.4 (95% CI −8.3 to 1.6), meaning that there was no statistically significant difference between both groups.

### ICU admission (hypertensive patients)

#### ACEIs/ARBs

**Felice** [3] found that admission to semi-intensive/intensive care units was less likely to occur in hypertensive patients on ARBs or ACEIs (adjusted OR 0.25, 95% CI 0.09–0.66, *p* = 0.006).

**Selçuk** [7] found a statistically significant difference (*p* = 0.001) between ICU admission for hypertensive ACEI/ARB users (50%) compared with hypertensive non-users (17.9%). No correction for confounders was applied.

**Reynolds** [2] (included in the review by Mackey) studied the relation between ACEI or ARB use in hypertensive patients and the composite outcome ‘severe COVID-19’, defined as ICU admission, use of noninvasive or mechanical ventilation, or death. The mean difference between users and non-users of this medication was −0.5 (95% CI −4.3 to 3.2).

One study found no statistically significant difference, one study found a statistically significant difference in favour of ARB/ACEI users and one study found a statistically significant difference in favour of non-users.

#### ARBs

**Reynolds** [2] (included in the review by Mackey) studied the relation between ARB use in hypertensive patients and the composite outcome ‘severe COVID-19’, defined as ICU admission, use of noninvasive or mechanical ventilation, or death. The mean difference between users and non-users of this medication was 0.1 (95% CI −4.8 to 4.9), meaning there was no statistically significant difference.

#### ACEIs

**Reynolds** [2] (included in the review by Mackey) studied the relation between ACEI use in hypertensive patients and the composite outcome ‘severe COVID-19’, defined as ICU admission, use of noninvasive or mechanical ventilation, or death. The mean difference between users and non-users of this medication was −3.3 (−8.2 to 1.7), meaning there was no statistically significant difference.

#### Thiazolidinediones

**Reynolds** [2] studied the relation between thiazide diuretics and the composite outcome ‘severe COVID-19’, defined as ICU admission, use of noninvasive or mechanical ventilation, or death. The mean difference between users and non-users of this medication was 0.6 (95% CI −4.5 to 5.7), meaning there was no statistically significant difference.

### Hospital admission (overall group)

#### ACEIs/ARBs

**López-Otero** [6] reported 731 patients admitted to the hospital (75.8%), of which 210 were ACEI/ARB users. They concluded in a multivariate analysis (adjusted for days with symptoms, fever, arterial oxygen saturation, < 95%, age, sex, health personnel, institutionalised, dependency status, dementia, hypertension, dyslipidaemia, ventricular dysfunction, lung disease, previous cancer, hypothyroidism and antiplatelet therapy) that there was no statistically significant difference in hospital admission in ACEI/ARB users vs non-users (OR 0.85, 95% CI 0.45–1.64, *p* = 0.638).

**Rentsch** [2] (included in the review by Mackey) found in a multivariate analysis that there was no statistically significant difference between hospital admission in ACEI/ARB users vs non-users (adjusted OR 1.24, 95% CI 0.79–1.95). This study was not peer-reviewed.

Two studies found no statistically significant difference (one of those studies was not peer-reviewed).

#### ARBs

**López-Otero** [6] concluded in a multivariate analysis (adjusted for days with symptoms, fever, arterial oxygen saturation < 95%, age, sex, health personnel, institutionalised, dependency status, dementia, hypertension, dyslipidaemia, ventricular dysfunction, lung disease, previous cancer, hypothyroidism and antiplatelet therapy) that there was no statistically significant difference in hospital admission in ARB users (*n* = 50) vs non-users (*n* = 134) (OR 1.10, 95% CI 0.59–2.04 *p* = 0.757).

#### ACEIs

**Rossi** [2] (included in the review by Mackey) performed a multivariate analysis and found an adjusted hazard ratio (HR) with ACEIs (adjusted for age, sex and Charlson Comorbidity Index score) of 1.13 (95% CI 1.1–1.5). When the analysis was restricted to patients with cardiovascular disease, the adjusted HR was 1.12 (95% CI 0.82–1.54, adjusted for age, sex and Charlson Comorbidity Index score).

**López-Otero** [6] concluded in a multivariate analysis (adjusted for days with symptoms, fever, arterial oxygen saturation < 95%, age, sex, health personnel, institutionalised, dependency status, dementia, hypertension, dyslipidaemia, ventricular dysfunction, lung disease, previous cancer, hypothyroidism and antiplatelet therapy) that there was no statistically significant difference in hospital admission in ACEI users (*n* = 20) vs non-users (*n* = 77) (OR 0.78, 95% CI 0.38–1.60, *p* = 0.505).

### Hospital admission (hypertensive patients)

#### ACEIs/ARBs

**Felice** [3] concluded in a multivariate analysis (adjusted for gender, BMI, days with symptoms prior to admission, previous cardiovascular events, diabetes and cancer) that there was no statistically significant difference in hospital admission in hypertensive ACEI/ARB users vs non-users (OR 0.39, 95% CI 0.05–2.94, *p* = 0.365).

### Length of stay (hypertensive patients)

#### ACEI/ARBS

**Selçuk** [7] assessed length of stay for hypertensive patients on ACEIs/ARBS and hypertensive patients on other medication. There was no statistically significant difference between the hypertensive patients on ACEIs/ARBs and those not using ACEIs/ARBs: 9 days ± 6, non-users 8 days ± 4 (*p* = 0.524).

**Zhou** [9] found no statistically significant difference (*p* = 0.405) in hospital length of stay in hypertensive patients using ACEIs/ARBs (mean 10.1 days, SD 5.2) and hypertensive patients using other antihypertensive drugs (mean 11.7, SD 6.0). We calculated the mean difference between the groups, which was 1.60 (95% CI‑2.31–5.51).

Two studies found no statistically significant difference.

### Ventilation (overall group)

Considering the outcome of ventilation it should be noted that ventilation was defined differently in each study. **Jung** [5] assessed mechanical ventilation, **Felice** [3] assessed oxygen therapy and non-invasive ventilation, **Gao** [4] assessed invasive mechanical ventilation, **Mancia** [2](included in the review by Mackey) assisted ventilation and **Selçuk** [7] endotracheal intubation.

#### ACEIs/ARBs

**Reynolds** [2] (included in the review by Mackey) studied the relation between ACEI or ARB use and the composite outcome ‘severe COVID-19’, defined as ICU admission, use of noninvasive or mechanical ventilation, or death. The mean difference between users and non-users of this medication was −0.1 (95% CI −3.7 to 3.5), meaning that there was no statistically significant difference between both groups.

#### ARBs

**Reynolds** [2] (included in the review by Mackey) studied the relation between ARBs and the composite outcome ‘severe COVID-19’, defined as ICU admission, use of noninvasive or mechanical ventilation, or death. The mean difference between users and non-users of this medication was −1.9 (95% CI −6.6 to 2.8), meaning that there was no statistically significant difference between both groups.

**Jung** [5] studied the relation between ACEI/ARB use and ventilation (mechanical ventilation). Since most of the included patients were only taking ARBs, the results of this paper are included in the ARBs-only category. Jung calculated an adjusted OR of 1.03 (95% CI 0.50–2.13, *p* = 0.93).

**Mancia** [2] (included in the review by Mackey) studied the relation between ARBs and the composite outcome ‘severe COVID-19’ defined as assisted ventilation or death. The adjusted OR was 0.83 (95% CI 0.63–1.10).

Three studies found no statistically significant difference.

#### ACEIs

**Reynolds** [2] (included in the review by Mackey) studied the relation between ACEIs and the composite outcome ‘severe COVID-19’, defined as ICU admission, use of noninvasive or mechanical ventilation, or death. The mean difference between users and non-users of this medication was −1.9 (95% CI −6.6 to 2.8), meaning that there was no statistically significant difference between both groups.

**Mancia** [2] (included in the review by Mackey) studied the relation between ACEIs and the composite outcome ‘severe COVID-19’ defined as assisted ventilation or death. The adjusted OR was 0.91 (95% CI 0.69–1.21).

Two studies found no statistically significant difference.

#### Thiazolidinediones

**Reynolds** [2] studied the relation between thiazide diuretics and the composite outcome ‘severe COVID-19’, defined as ICU admission, use of noninvasive or mechanical ventilation, or death. The mean difference between users and non-users of this medication was −3.4 (95% CI −8.3 to 1.6), meaning that there was no statistically significant difference between both groups.

### Ventilation (hypertensive patients)

#### ACEIs/ARBs

**Gao** [4] compared users of RAAS inhibitors (5, 2.7%) with non-RAAS inhibitor users (25, 4.7%) and found no statistically significant difference (*p* = 0.292). **Felice** [3] found an adjusted OR of 0.58, 95% CI 0.21–1.60, *p* = 0.296. **Selçuk** [7] reported a statistically significant difference between ACEI/ARB users (44.6% required ventilation) vs non-users (10.3% required ventilation) (*p* < 0.001); however, there was no correction for confounders performed.

**Reynolds** [2] (included in the review by Mackey) studied the relation between ACEI or ARB use in hypertensive patients and the composite outcome ‘severe COVID-19’, defined as ICU admission, use of noninvasive or mechanical ventilation, or death. The mean difference between users and non-users of this medication was −0.5 (95% CI −4.3 to 3.2).

#### ARBs

**Reynolds** [2] (included in the review by Mackey) studied the relation between ARB use in hypertensive patients and the composite outcome ‘severe COVID-19’, defined as ICU admission, use of noninvasive or mechanical ventilation, or death. The mean difference between users and non-users of this medication was 0.1 (95% CI −4.8 to 4.9), meaning that there was no statistically significant difference between both groups.

#### ACEIs

**Reynolds** [2] (included in the review by Mackey) studied the relation between ACEI use in hypertensive patients and the composite outcome ‘severe COVID-19’, defined as ICU admission, use of noninvasive or mechanical ventilation, or death. The mean difference between users and non-users of this medication was −3.3 (−8.2 to 1.7), meaning that there was no statistically significant difference between both groups.

#### Thiazolidinediones

**Reynolds** [2] studied the relation between thiazide diuretics and the composite outcome ‘severe COVID-19’, defined as ICU admission, use of noninvasive or mechanical ventilation, or death. The mean difference between users and non-users of this medication was 0.6 (95% CI −4.5 to 5.7), meaning that there was no statistically significant difference between both groups.

### Thromboembolic complications (overall)

#### ACEIs/ARBs

**López-Otero** [6] reported on heart failure (defined according to the European Society of Cardiology guidelines). In a multivariate analysis, there was no statistically significant difference between ACEI/ARB users and non-users (OR 1.37, 95% CI 0.39–4.77, *p* = 0.622). The absence of an impact on heart failure remained both in the multivariate analysis and in the propensity score model, including in the evaluation of treatment taken for more than 1 year.

#### ARBs

**Jung** [5] reported on acute cardiac events defined as cardiac arrest, myocardial infarction or acute heart failure. Since most of the included patients were only taking ARBs, the results of this paper are included in the ARBs-only category. No statistically significant differences were observed between RAAS inhibitor users and non-users in terms of acute cardiac events (OR 0.88, 95% CI 0.59 1.31, *p* = 0.53).

**López-Otero** [6] reported on heart failure (defined according to the European Society of Cardiology guidelines). In a multivariate analysis, there was no statistically significant difference between ARB users and non-users (OR 0.46, 95% CI 0.12–1.72 *p* = 0.248).

#### ACEIs

**López-Otero** [6] reported on heart failure (defined according to the European Society of Cardiology guidelines). In a multivariate analysis, there was no statistically significant difference between ACEI users and non-users (OR 3.01, 95% CI 0.89–10.16 *p* = 0.076).

## Level of evidence of the literature

The level of evidence was assessed according to the GRADE methodology (GRADE: Grading Recommendations Assessment, Development and Evaluation, http://www.gradeworkinggroup.org/).

### Mortality (overall group)

#### ACEIs/ARBs

Starting with a high level of evidence for observational studies (prognostic question), the level of evidence regarding the outcome of mortality was downgraded by one level because of risk of bias (not all studies corrected for confounders, number of events sometimes not reported) to ‘moderate’.

#### ARBs

Starting with a high level of evidence for observational studies (prognostic question), the level of evidence regarding the outcome of mortality was downgraded by one level because of the risk of bias (some patients were still hospitalised at the moment of analysis and may have later reached the outcome of mortality) and one level for indirectness (two studies used a composite outcome measure) to ‘low’.

#### ACEIs

Starting with a high level of evidence for observational studies (prognostic question), the level of evidence regarding the outcome of mortality was downgraded by one level because of risk of bias (one study not peer reviewed, some patients were still hospitalised at the moment of analysis and may have later reached the outcome of mortality), one level because of indirectness (two studies used a composite outcome measure and one study assessed 7‑day mortality) to ‘low’.

#### NSAIDs

Starting with a high level of evidence for observational studies (prognostic question), the level of evidence regarding the outcome of mortality was downgraded by one level because of risk of bias (unable to assess if the groups are comparable and information was unavailable on how many patients were still hospitalised at the moment of analysis) and two levels for imprecision (only one study available, small number of patients included) to ‘very low’.

#### Thiazolidinediones

Starting with a high level of evidence for observational studies (prognostic question), the level of evidence regarding the outcome of mortality was downgraded by one level because of risk of bias (some patients were still hospitalised at the moment of analysis and may have later reached the outcome of mortality) and one level for indirectness (a composite outcome measure was used) to ‘low’.

### Mortality (hypertensive patients)

#### ACEIs/ARBs

Starting with a high level of evidence for observational studies (prognostic question), the level of evidence regarding the outcome of mortality was downgraded by one level because of risk of bias (number of events sometimes not reported but only ORs), and one level for imprecision (difference in effect size) to ‘low’.

#### ARBs

Starting with a high level of evidence for observational studies (prognostic question), the level of evidence regarding the outcome of mortality was downgraded by one level because of risk of bias (some patients were still hospitalised at the moment of analysis and may have later reached the outcome of mortality), and one level because of indirectness (one study used a composite outcome measure) to ‘low’.

#### ACEIs

Starting with a high level of evidence for observational studies (prognostic question), the level of evidence regarding the outcome of mortality was downgraded by one level because of risk of bias (some patients were still hospitalised at the moment of analysis and may have later reached the outcome of mortality), one level for imprecision (some studies showed a statistically significant difference and some did not) and one level because of indirectness (a composite outcome measure was used) to ‘very low’.

#### Thiazolidinediones

Starting with a high level of evidence for observational studies (prognostic question), the level of evidence regarding the outcome of mortality was downgraded by one level because of risk of bias (some patients were still hospitalised at the moment of analysis and may have later reached the outcome of mortality) and one level because of indirectness (a composite outcome measure was used) to ‘low’.

### ICU admission (overall group)

#### ACEIs/ARBs

Starting with a high level of evidence for observational studies (prognostic question), the level of evidence regarding the outcome of ICU admission was downgraded by one level because of risk of bias (one study not peer reviewed, follow-up duration unclear), one level because of imprecision (some studies showed no effect, one study showed a significant effect) and one level because of indirectness (in one study a composite outcome measure was used) to ‘very low’.

#### ARBs

Starting with a high level of evidence for observational studies (prognostic question), the level of evidence regarding the outcome of ICU admission was downgraded by one level because of risk of bias (some patients were still hospitalised at the moment of analysis and could still be admitted to the ICU at a later stage), one level for indirectness (one study used a composite outcome measure) and one level for imprecision (small number of events) to ‘very low’.

#### ACEIs

Starting with a high level of evidence for observational studies (prognostic question), the level of evidence regarding the outcome of ICU admission was downgraded by one level because of risk of bias (some patients were still hospitalised at the moment of analysis and could still be admitted to the ICU at a later stage), one level for indirectness (one study used a composite outcome measure) and one level because of imprecision (low number of events) to ‘very low’.

#### Thiazolidinediones

Starting with a high level of evidence for observational studies (prognostic question), the level of evidence regarding the outcome of ICU admission was downgraded by one level because of risk of bias (some patients were still hospitalised at the moment of analysis and could still be admitted to the ICU at a later stage) and one level because of indirectness (a composite outcome measure was used) to ‘low’.

### ICU admission (hypertensive patients)

#### ACEIs/ARBs

Starting with a high level of evidence for observational studies (prognostic question), the level of evidence regarding the outcome of ICU admission was downgraded by one level because of risk of bias and one level for imprecision (wide range of effects) and one level because of indirectness (a composite outcome measure was used in one study and in one study the outcome was semi-intensive care or intensive care) to ‘very low’.

#### ARBs

Starting with a high level of evidence for observational studies (prognostic question), the level of evidence regarding the outcome of ICU admission was downgraded by one level because of risk of bias (some patients were still hospitalised at the moment of analysis and could still be admitted to the ICU at a later stage) and one level because of indirectness (a composite outcome measure was used) to ‘low’.

#### ACEIs

Starting with a high level of evidence for observational studies (prognostic question), the level of evidence regarding the outcome of ICU admission was downgraded by one level because of risk of bias (some patients were still hospitalised at the moment of analysis and could still be admitted to the ICU at a later stage) and one level because of indirectness (a composite outcome measure was used) to ‘low’.

#### Thiazolidinedione*s*

Starting with a high level of evidence for observational studies (prognostic question), the level of evidence regarding the outcome of ICU admission was downgraded by one level because of risk of bias (some patients were still hospitalised at the moment of analysis and could still be admitted to the ICU at a later stage) and one level because of indirectness (a composite outcome measure was used) to ‘low’.

### Hospital admission (overall)

#### ACEIs/ARBs

Starting with a high level of evidence for observational studies (prognostic question), the level of evidence regarding the outcome of hospital admission was downgraded by two levels because of risk of bias (one study not peer reviewed, duration of follow-up unclear) to ‘low’.

#### ARBs

Starting with a high level of evidence for observational studies (prognostic question), the level of evidence regarding the outcome of hospital admission was downgraded by one level because of risk of bias (follow-up duration unclear), and one level for imprecision (small number of events) to ‘low’.

#### ACEIs

Starting with a high level of evidence for observational studies (prognostic question), the level of evidence regarding the outcome of hospital admission was downgraded by one level because of risk of bias (follow-up duration unclear), and one level for imprecision (small number of events, 2 studies showed conflicting results) to ‘low’.

### Hospital admission (hypertensive patients)

#### ACEIs/ARBs

Starting with a high level of evidence for observational studies (prognostic question), the level of evidence regarding the outcome of hospital admission was downgraded by one level because of risk of bias (the study only included hypertensive subjects who presented to the emergency department with acute respiratory symptoms/fever), and two levels because of imprecision (small sample size, wide confidence interval) to ‘very low’.

### Length of stay (overall)

#### ACEIs/ARBs

Starting with a high level of evidence for observational studies (prognostic question), the level of evidence regarding the outcome of length of stay was downgraded by two levels because of risk of bias (no correction for confounders, in one study follow-up duration unclear) and one level because of imprecision (small number of included patients) to ‘very low’.

### Ventilation (overall group)

#### ACEIs/ARBs

Starting with a high level of evidence for observational studies (prognostic question), the level of evidence regarding the outcome of ventilation was downgraded by one level because of risk of bias (some patients may not have reached the outcome by the date of analysis) and one level because of indirectness (a composite outcome measure was used) to ‘low’.

#### ARBs

Starting with a high level of evidence for observational studies (prognostic question), the level of evidence regarding the outcome of ventilation was downgraded by one level because of risk of bias (some patients may not have reached the outcome by the date of analysis), one level for imprecision (wide confidence interval) and one level because of indirectness (a composite outcome measure was used in two studies) to ‘very low’.

#### ACEIs

Starting with a high level of evidence for observational studies (prognostic question), the level of evidence regarding the outcome of ventilation was downgraded by one level because of risk of bias (some patients may not have reached the outcome by the date of analysis), one level for imprecision (wide confidence interval) and one level because of indirectness (a composite outcome measure was used in two studies) to ‘very low’.

#### Thiazolidinediones

Starting with a high level of evidence for observational studies (prognostic question), the level of evidence regarding the outcome of ventilation was downgraded by one level because of risk of bias (some patients may not have reached the outcome by the date of analysis) and one level because of indirectness (a composite outcome measure was used) to ‘low’.

### Ventilation (hypertensive patients)

#### ACEIs/ARBs

Starting with a high level of evidence for observational studies (prognostic question), the level of evidence regarding the outcome of ventilation was downgraded by one level because of risk of bias (some studies did not correct for confounding), one level for imprecision (some studies described a significant difference, some studies found no significant difference) and one level because of indirectness (composite outcome measure, different definitions of ventilation) to ‘very low’.

#### ARBs

Starting with a high level of evidence for observational studies (prognostic question), the level of evidence regarding the outcome of ventilation was downgraded by one level because of risk of bias (some patients may not have reached the outcome by the date of analysis) and one level because of indirectness (a composite outcome measure was used) to ‘low’.

#### ACEIs

Starting with a high level of evidence for observational studies (prognostic question), the level of evidence regarding the outcome of ventilation was downgraded by one level because of risk of bias (some patients may not have reached the outcome by the date of analysis) and one level because of indirectness (a composite outcome measure was used) to ‘low’.

#### Thiazolidinediones

Starting with a high level of evidence for observational studies (prognostic question), the level of evidence regarding the outcome of ventilation was downgraded by one level because of risk of bias (some patients may not have reached the outcome by the date of analysis) and one level because of indirectness (a composite outcome measure was used) to ‘low’.

### Thromboembolic complications (overall)

#### ACEIs/ARBs

Starting with a high level of evidence for observational studies (prognostic question), the level of evidence regarding the outcome of thromboembolic complications was downgraded by one level because of risk of bias (some patients were still hospitalised at the moment of analysis and could still develop thromboembolic complications), one level because of indirectness (thromboembolic complications defined as heart failure) and one level because of imprecision (small number of events) to ‘very low’.

#### ARBs

Starting with a high level of evidence for observational studies (prognostic question), the level of evidence regarding the outcome of thromboembolic complications was downgraded by one level because of risk of bias (some patients were still hospitalised at the moment of analysis and could still develop thromboembolic complications), one level because of indirectness (thromboembolic complications defined as heart failure in one study and in one study a small number of patients in the group were receiving ARBs) and one level because of imprecision (small number of events) to ‘very low’.

#### ACEIs

Starting with a high level of evidence for observational studies (prognostic question), the level of evidence regarding the outcome of thromboembolic complications was downgraded by one level because of risk of bias (some patients were still hospitalised at the moment of analysis and could still develop thromboembolic complications), one level because of indirectness (thromboembolic complications defined as heart failure) and one level because of imprecision (wide confidence interval) to ‘very low’.

## Conclusion

### Mortality (overall group)

ACEI/ARB use probably does not increase mortality. *Sources: Zhang, Imam, López-Otero, Reynolds* **(moderate GRADE level)***.*

The evidence is uncertain about the effect of ACEI use on mortality. *Source: Mackey* (**low GRADE level**).

The evidence suggests that ARB use does not increase mortality. *Sources: Reynolds, Mancia, Jung, Mehra* **(low GRADE level)**.

The evidence suggests that thiazolidinedione use does not increase mortality. *Source: Mackey* (**low GRADE level**).

The evidence is very uncertain about the effect of NSAID use on mortality. *Source: Imam* (**very low GRADE level**).

### Mortality (hypertensive patients)

ACEI/ARB use may not increase mortality, but the evidence is very uncertain. *Sources: Zhang, Felice, Gao, Zhou, Reynolds, Selçuk * (**very low GRADE level**).

The evidence suggests that ARB or thiazolidinedione use does not increase mortality. *Sources: Reynolds, Mehra * (**low GRADE level**).

The evidence is very uncertain about the effect of ACEI use on mortality. *Sources: Reynolds, Mehra * (**low GRADE level**).

### ICU admission (overall group)

The evidence is very uncertain about the effect of ACEI/ARB or ACEI use on ICU admission. *Sources: Reynolds, López-Otero, Bean, Rentsch * (**very low GRADE level**).

ARB use may have no effect on ICU admission, but the evidence is very uncertain. *Sources: Reynolds, López-Otero * (**very low GRADE level**).

The evidence suggests that thiazolidinedione use does not increase ICU admission. *Sources: Reynolds * (**low GRADE level**).

### ICU admission (hypertensive patients)

The evidence is very uncertain about the effect of ACEIs/ARBs on ICU admission. *Sources: Reynolds, Felice, Selçuk * (**very low GRADE level**).

The evidence suggests that the use of ARBs, ACEIs and thiazolidinediones does not increase ICU admission. *Source: Reynolds * (**low GRADE level**).

### Hospital admission (overall group)

The evidence suggests that the use of ACEIs/ARBs, ARBs and ACEIs does not increase hospital admission. *Sources: López-Otero, Rentsch, Rossi* (**low GRADE level**).

### *Hospital admission (hypertensive patients*)

ACEI/ARB use may have no effect on hospital admission, but the evidence is very uncertain. *Source: Felice * (**very low GRADE level**).

### Length of stay (hypertensive patients)

ACEIs/ARBs may have no effect on hospital length of stay, but the evidence is very uncertain. *Sources: Selçuk, Zhou * (**very low GRADE level**).

### Ventilation (overall group)

The evidence suggests that the use of ACEIs/ARBs, ACEIs and thiazolidinediones does not increase the need for ventilation. *Sources: Reynolds, Jung, Mancia * (**low GRADE level**).

ARB use may have no effect on ventilation, but the evidence is very uncertain.

*Sources: Reynolds, Mancia, Jung * (**very low GRADE level**).

### Ventilation (hypertensive patients)

The evidence is very uncertain about the effect of ACEI/ARB use on ventilation. *Sources: Gao, Reynolds, Felice, Selçuk * (**very low GRADE level**).

The evidence suggests that use of ARBs, ACEIs and thiazolidinediones does not increase the need for ventilation. *Source: Reynolds * (**low GRADE level**).

### Thromboembolic complications (overall group)

Use of ACEIs/ARBs, ARBs and ACEIs may have no effect on thromboembolic complications, but the evidence is very uncertain. *Sources: López-Otero, Jung * (**very low GRADE level**).

## Discussion

The quality of the evidence from the included studies is generally low to very low. In a new situation (such as COVID-19) it is logical that most studies cannot yet meet the strict requirements set for high-quality research. However, the GRADE method compares the quality of the evidence against the best possible quality and not against the best possible quality in the current situation. The GRADE system reflects confidence in the estimation of the effect of an intervention. When the modules and search are updated, better quality studies will hopefully become available and the level of quality of evidence can be adjusted accordingly.

In this literature summary, the effect of the use of ACEIs, ARBs, NSAIDs and thiazolidinediones in COVID-19 patients on the outcome measure of mortality was investigated, more specifically admission, hospitalisation, length of stay, ventilation and thromboembolic complications. Mortality was defined as a critical outcome measure. The literature shows that there is no association between the use of ACEIs/ARBs (combined or separately) with mortality for both the overall group (comparing users with non-users) and the subgroup with only hypertensive patients. The quality of the evidence varies from moderate (for the overall group in which ACEI/ARB users combined) to very low. With regard to NSAID use, the quality of the scientific evidence is very low and no conclusions can be drawn for practice.

For the important outcome measures, the quality of the evidence was low to very low. Again, the use of ACEIs, ARBs (both alone and combined) and thiazolidinediones indicates little or no association with the important outcome measures.

There are hardly any studies in which use of ACEIs or ARBs was associated with a higher risk of poor outcome, one of the few (Sulcuk et al.[7]) was a very small study. In general, there was a weak association with better outcomes with the use of these agents (Figs. 1 and 2). This virtually excludes that these agents have a negative effect on the course of a COVID-19 infection. It does not show that they are protective, but a protective effect cannot be ruled out. Repeated exclusion of association with adverse outcomes is, however, able to assess the likelihood that such a drug will have direct adverse effects as very unlikely. In summary, there is strong evidence for the absence of a relationship between ACEI use, ARB use and worse outcomes. There is no strong evidence for the protective effects of these agents, but this cannot be ruled out either.

### CAPACITY registry

CAPACITY (Cardiac complicAtions in Patients with SARS Corona vIrus 2 regisTrY) is an international registry of patients with COVID-19 based on the ISARIC-WHO CRF, supplemented with information on specific cardiovascular parameters (https://capacity-covid.eu/) [10]. CAPACITY started in Spring 2020 and in August 2020, 61 hospitals from 13 countries contributed to the data collection. CAPACITY contains extensive information about patients with COVID because approximately 40% of the COVID-19 patients admitted to the Netherlands are included in the registry (*n* = 5524).

The peer-reviewed publication of CAPACITY on the topic of this module is currently in preparation. Therefore, the results of CAPACITY cannot yet be included in the literature search, but the preliminary results of CAPACITY are included in the considerations. The peer-reviewed publication on the subject of this module is expected shortly and an update of the module will include the publication in the literature search.

The first analyses of the CAPACITY data are in line with the above findings. In addition, very preliminary analyses seem to suggest that discontinuation of more powerful ARBs is associated with worse outcomes, even after adjustment for confounders. These analyses have yet to be definitively confirmed.

### Recommendations

#### Rationale of the recommendation: weighting of arguments for and against the interventions

There is no relationship between ACEI and ARB use and worse outcomes for COVID-19 infection. This is a strong fact given that several studies do not find this association, except for one small study. Earlier use of ACEIs/ARBs appears to be associated with less adverse outcomes of severe COVID-19 infection. Discontinuation of ACEIs/ARBs during admission for COVID-19 infection is associated with worse outcomes. The associations found are weak, but with a reasonable effect size. The type of evidence is also weak (retrospective). (See Box 1).

##### Box 1 Recommendations

- Do not discontinue or discourage ACEIs/ARBs use in patients experiencing COVID-19 infection of any severity of infection, other than for acute haemodynamic reasons, acute severe renal impairment, or severe renal insufficiency

### Gaps in evidence

There is a knowledge gap regarding the effects of previous use of NSAIDs. Due to a lack of studies on this subject, no recommendations can now be formulated. No formal study has been conducted into the effect of continuing or discontinuing ACEIs/ARBs during admission. In addition, it is often not reported whether ACEIs/ARBs are continued or are discontinued during admission.

**Table 1:** General study characteristics

Author (year)	Study type	Comments	*N*	Country	Outcome
Zhang [1]	Systematic review and meta-analysis			Multiple	Mortality
Mackey [2]	Systematic review	There is overlap between studies included in Zhang and Mackey. Mackey included two studies for mortality that are not included in Zhang. One of those papers is not peer-reviewed and the other is in Chinese, so neither can be used		Multiple	Mortality was assessed but not used, see comments ICU admission Hospital admission
Felice [3]	Observational study		133	Italy	Mortality, ICU admission, hospital admission, ventilation
Gao [4]	Observational study		2877	China	Mortality, ventilation
Imam [8]	Observational study		1305	US	Mortality
Jung [5]	Observational study		5179	Korea	Mortality, ventilation, thromboembolic complications
López-Otero [6]	Observational study		965	Spain	Mortality, ICU admission, hospital admission, thromboembolic complications
Selçuk [7]	Observational study		113	Turkey	Mortality, ICU admission, hospital admission, ventilation, length of stay
Zhou [9]	Observational study		110 (36 of which were used for the analysis of interest)	China	Mortality, length of stay

## Supplementary Information

**Table S1 **Literature search strategy

**Table S2 **Excluded studies

**Table S3** Evidence table for systematic review of RCTs and observational studies (intervention studies)

**Table S4** Evidence table for intervention studies (randomised controlled trials and non-randomised observational studies [cohort studies, case-control studies, case series])

**Table S5 **Quality assessment for systematic reviews of RCTs and observational studies Based on AMSTAR checklist (Shea et al.; 2007, BMC Methodol 7: 10; https://doi.org/10.1186/1471-2288-7-10) and PRISMA checklist (Moher et al. 2009, PLoS Med 6: e1000097; https://doi.org/10.1371/journal.pmed1000097)

**Table S6** Risk of bias for intervention studies (observational: non-randomised clinical trials, cohort and case-control studies)
