# Supplementary material for: The effects of ACE2 expression mediating pharmacotherapy in COVID-19 patients
Source: Neth Heart J. 2021 Apr 16;29(Suppl 1):20–34. doi: 10.1007/s12471-021-01573-8 (PMC8050813; doi:10.1007/s12471-021-01573-8)
Supplement: Supplementary file 1 — Table S1 Literature search strategy [file 12471_2021_1573_MOESM1_ESM.docx]

**Table S1** Literature search strategy

**OVID/Medline**

1 exp angiotensin converting enzyme inhibitors/ or exp Anti-Inflammatory Agents, Non-Steroidal/ or exp Angiotensin Receptor Antagonists/ or exp Thiazolidinediones/ (265060)

2 (angiotensin ii receptor antagonist* or angiotensin ii receptor blocker* or angiotensin ii receptor blocking agent* or angiotensin receptor antagonist* or angiotensin receptor blocker* or angiotensin receptor blocking agent* or arb or nsaid* or thiazol* or ace inhibitor* or angiotensin converting enzyme inhibiting agent* or angiotensin converting enzyme inhibitor* or angiotensin i converting enzyme inhibitor* or dipeptidyl carboxypeptidase i inhibitor* or dipeptidyl carboxypeptidase inhibitor* or kininase ii inhibitor* or peptidyl dipeptidase inhibitor* or peptidyldipeptide hydrolase inhibitor* or acei).ti,ab,kf. (87285)

3 1 or 2 (304951)

4 ((exp Coronavirus/ or Coronavirus Infections/ or pneumonia virus*.ti,ab,kf. or cov.ti,ab,kf.) and ((outbreak or wuhan).ti,ab,kf. or novel.af. or '19'.ti,ab,kf. or '2019'.ti,ab,kf. or epidem*.af. or epidemy.af. or epidemic*.af. or pandem*.af. or new.ti,ab,kf.)) or (coronavirus* or 'corona virus*' or ncov or '2019ncov' or 'covid19' or "covid 19" or "sars cov 2" or 'sars2' or "ncov 2019" or "sars coronavirus 2" or "sars corona virus 2" or "severe acute respiratory syndrome cov 2" or "severe acute respiratory syndrome cov2" or "severe acute respiratory syndrome cov*").ti,ab,kf. (40060)

5 3 and 4 (301)

6 limit 5 to dt="20191201-20220101" (276)

7 6 not ((exp animals/ or exp models, animal/) not humans/) not (letter/ or comment/ or editorial/) (213)

**Embase**

| No. | Query | Results |
| --- | --- | --- |
| #4 | #3 NOT ('conference abstract'/it OR 'editorial'/it OR 'letter'/it OR 'note'/it) NOT (('animal experiment'/exp OR 'animal model'/exp OR 'nonhuman'/exp) NOT 'human'/exp) | **296** |
| #3 | #1 AND #2 | **427** |
| #2 | 'angiotensin receptor antagonist'/exp OR 'angiotensin ii receptor antagonist*':ti,ab,kw OR 'angiotensin ii receptor blocker*':ti,ab,kw OR 'angiotensin ii receptor blocking agent*':ti,ab,kw OR 'angiotensin receptor antagonist*':ti,ab,kw OR 'angiotensin receptor blocker*':ti,ab,kw OR 'angiotensin receptor blocking agent*':ti,ab,kw OR arb:ti,ab,kw OR 'nonsteroid antiinflammatory agent'/exp OR nsaid*:ti,ab,kw OR 'thiazolidine derivative'/exp OR 'thiazol*':ti,ab,kw OR 'dipeptidyl carboxypeptidase inhibitor'/exp OR 'ace inhibitor*':ti,ab,kw OR 'angiotensin converting enzyme inhibiting agent*':ti,ab,kw OR 'angiotensin converting enzyme inhibitor*':ti,ab,kw OR 'angiotensin i converting enzyme inhibitor*':ti,ab,kw OR 'dipeptidyl carboxypeptidase i inhibitor*':ti,ab,kw OR 'dipeptidyl carboxypeptidase inhibitor*':ti,ab,kw OR 'kininase ii inhibitor*':ti,ab,kw OR 'peptidyl dipeptidase inhibitor*':ti,ab,kw OR 'peptidyldipeptide hydrolase inhibitor*':ti,ab,kw OR acei:ti,ab,kw | **965640** |
| #1 | ('coronavirus disease 2019'/exp OR (('coronavirinae'/exp OR 'coronavirus infection'/de OR coronavirus*:ti,ab,kw OR 'corona virus*':ti,ab,kw OR 'pneumonia virus*':ti,ab,kw OR cov:ti,ab,kw OR ncov:ti,ab,kw) AND (outbreak:ti,ab,kw OR wuhan:ti,ab,kw)) OR covid19:ti,ab,kw OR 'covid 19':ti,ab,kw OR ((coronavirus*:ti,ab,kw OR 'corona virus*':ti,ab,kw) AND 2019:ti,ab,kw) OR 'sars cov 2':ti,ab,kw OR sars2:ti,ab,kw OR 'coronavirus*':ti,ab,kw OR 'corona virus*':ti,ab,kw OR 'ncov 2019':ti,ab,kw OR ncov:ti,ab,kw OR 'sars coronavirus 2':ti,ab,kw OR 'sars corona virus 2':ti,ab,kw OR 'severe acute respiratory syndrome cov 2':ti,ab,kw OR 'severe acute respiratory syndrome cov2':ti,ab,kw) AND [2019-2020]/py | **21099** |
