# Supplementary material for: The effects of ACE2 expression mediating pharmacotherapy in COVID-19 patients
Source: Neth Heart J. 2021 Apr 16;29(Suppl 1):20–34. doi: 10.1007/s12471-021-01573-8 (PMC8050813; doi:10.1007/s12471-021-01573-8)
Supplement: Supplementary file 2 — Table S2 Excluded studies [file 12471_2021_1573_MOESM2_ESM.docx]

**Table S2** Excluded studies

| **Author and year** | **Reason for exclusion** |
| --- | --- |
| de Abajo (2020) | Wrong C: C are not COVID-19 patients |
| Grover (2020) | Search strategy unclear. The authors state a quality assessment was performed but the results are not available |
| Guo (2020) | This is a research letter not a research paper, does not describe methods or results very well. Info regarding the characteristics of included studies is missing |
| Iaccarino (2020) | The authors analyse in a multilevel model the association with mortality, number of patients on ACE inhibitors not available. |
| Bean (2020) | Included in review Mackey |
| Cannata (2020) | Wrong comparison (continuation vs discontinuation), correspondence making it hard to assess methodology |
| Emilsson (2020) | Wrong O: outcome is serum level ace2 |
| Guo (2020) | Wrong I: use of medication was not a factor, not described in paper. Is included in one of the reviews |
| Huang (2020) | Wrong outcome |
| Khera (2020) | Wrong C: comparison of ACEi and ARBs users |
| Mancia (2020) | Included in review Mackey |
| Mehra (2020) | Included in review Zhang |
| Peng (2020) | Paper is in Chinese |
| Pirola (2020) | Wrong outcome: composite outcome (in-hospital death and/or severe illness) |
| Reynolds (2020) | Included in review Mackey |
| Tadic (2020) | Wrong study design: prevalence of hypertension and CVD. 2 studies included that match our PICO, but both these studies are already in the literature set. |
| Yang (2020) | Included in review Mackey |
| Zhang (2020) | Included in review Zhang |
| Johnson (2020) | Used publicly available country level data |
| Morales (2020) | Wrong comparison (no control group) |
| Spaak (2020) | Paper in Swedish |
| Zhang (2020) | Wrong intervention: statin with ACEI/ARB |
| Calò (2020)/Journal of hypertension | Is a correspondence, not a research paper including a method and result section. |
| Calò (2020)/Journal of medical virology | It is a letter to the editor not a research paper including a method and result section |
| Rauch (2020) | Wrong outcome and not a research paper including a method and result section, merely a discussion based on other (not necessarily covid) literature |
| Saber-Ayad (2020) | Description of literature on the topic but not systematic (no search strategy, no outcomes defined etc) |
| Sunden-Cullberg (2020) | This is a research letter not a research paper, does not describe methods or results very well |
| Tan (2020) | Wrong outcome (effects on the digestive system) |
| Bravi (2020) | Wrong outcome (severe COVID-19 and death is combined) |
| Timerbulatov (2020) | Paper not in English (Russian) |
| Li (2020) | Included in review Mackey |
| Talreja (2020) | This is a viewpoint that describes literature on the topic but is not a systematic literature overview (does not describe search strategy or a systematic description of outcomes) |
| Amat-Santos (2020) | Wrong study design: non-pre-specified interim analysis, only 11 COVID19 patients included |
| Autor Anonymous (2020); Title EMA advice on renin-angiotensin system  medicines during covid-19 pandemic | Wrong study design: no original study: summary of EMA advice |
| Chen (2020) | Wrong population: patients with diabetes and covid-19: focus on insulin, subgroup using ACE inhibitor (n=32) |
| Chodick (2020) | Wrong outcome: risk of COVID infection |
| de Abajo (2020) | same as rayyan-79961775 |
| Feng (2020) | Wrong study design: description of characteristics |
| Feng (2020) | same as rayyan-79961796 |
| Fosbol (2020) | Wrong outcome: risk of COVID infection |
| Gianfrancesco (2020) | Specifically focused on patients with rheumatic disease |
| Kolin (2020) | Wrong outcome |
| Mehta (2020) | Wrong outcome: risk of Covid infection |
| Rico-Mesa (2020) | Not a systematic evaluation of literature. Outcomes not defined |
| Singh (2020) | Harm or benefit in COVID-19 patients receiving RASB has not been typically assessed in the included studies yet. So intervention not present |
| Sriram (2020) | Wrong outcome: outcome in this study is ACE2 expression |
| Vaduganathan (2020) | No original data, no systematic review |
| Yang (2020) | Same as rayan 79962048 |
| Yousefifard (2020) | Wrong P, not COVID-19 patients |
| Zhang (2020) | Same as Rayan 79962053 |
| Zhou (2020) | Same as rayyan-79962061 |
| Kim (2020) | wrong outcome and analysis based on big data |
| Arjomandi Rad (2020) | Wrong outcome: outcome is thromboembolic events |
| Bidulka (2020) | Wrong P: no patients with COVID, wrong O: S. aureus |
| Russo (2020) | Aim of this study is to describe the prevalence of pre-admission antithrombotic therapies |
| Stafford (2020) | Wrong P (not covid patients), wrong outcome (pulmonary adverse drug events) |
|  |  |
