# Supplementary material for: The effects of ACE2 expression mediating pharmacotherapy in COVID-19 patients
Source: Neth Heart J. 2021 Apr 16;29(Suppl 1):20–34. doi: 10.1007/s12471-021-01573-8 (PMC8050813; doi:10.1007/s12471-021-01573-8)
Supplement: Supplementary file 3 — Table S3 Evidence table for systematic review of RCTs and observational studies (intervention studies) [file 12471_2021_1573_MOESM3_ESM.docx]

**Table S3** Evidence table for systematic review of RCTs and observational studies (intervention studies)

| **Study reference** | **Study characteristics** | **Patient characteristics** | **Intervention (I)** | **Comparison / control (C)** | **Follow-up** | **Outcome measures and effect size** | **Comments** |
| --- | --- | --- | --- | --- | --- | --- | --- |
| Zhang, 2020 | SR and meta-analysis of cohort and case-control studies  *Literature search between Jan 1, 2020 and May 9, 2020*  **A**: Feng, 2020  **B**: Li Juyi, 2020  **C**: Mancia, 2020  **D**: Meng, 2020  **E**: Reynolds, 2020  **F**: Tedeschi, 2020  **G**: Yang, 2020  **H**: Zhang, 2020  **I**: Mehra, 2020  **J**: Yu, 2020  **K**: Mehta, 2020  **L**: Li xiaochen et al, 2020  *Setting and country*  **A**: China  **B**: China  **C**: Italy  **D**: China  **E**: USA  **F**: Italy  **G**: China  **H**: China  **I**: Asia, Europe, and  North America  **J**: China  **K**: USA  **L**: China  Source of funding and conflicts of interest:  This review was supported by Zhejiang Provincial Natural Science  Foundation of China  Conflicts of interest: none declared | Inclusion criteria SR: (1) study design: case-control, case-crossover, self-controlled  case series (SCCS) or cohort study; (2) antihypertensive treatment: ACEI/ARB use versus non-ACEI/ARB use; (3) outcomes: the incidence of  COVID-19, critical cases, or death; (4)  Exclusion criteria SR: editorials, correspondences, conference abstracts and commentary articles  *12 studies included of which 8 report on mortality*  Important patient characteristics at baseline:  N (ACE/ARB; Non ACE/ARB), mean age  **A**: N= 33; N=80, 53y  **B**: N= 115; N=247, 66y  **C**: N (ACEI) = 1502 (ARB)= 1394; N/A, 68y  **D**: N= 17; N=25, 64y  **E**: N= 091; N= 986, 64y  **F**: N= 165; N=136, 76y  **G**: N= 43; N=48, 66y  **H**: N= 188; N= 940, 64y  **I**: N (ACEI) = 770 N (ARB)= 556; N (non-ACEI)=8140 N (nonARB)= 8354, 49y  **J**: N= 103; N=173, 60y  **K**: N (ACEI)= 116 N(ARB)=98; N(non-ACEI)=  1619  N(nonARB)= 1637, 49y  **L**: N= 42; N= 503, 60y  *Sex (% males):*  **A**: 57%  **B**: 52%  **C**: NA  **D**: 57%  **E**: 50%  **F**: 72%  **G**: 49%  **H**: 53%  **I**: 60%  **J**: 53%  **K**: 40%  **L**: 51%  Groups comparable at baseline?  This information is not available  *Confounder adjustment*  **A**: No  **B**: No  **C**: No  **D**: No  **E**: No  **F**: Age, gender, presence of CV comorbidities  and COPD  **G**: No  **H**: Age, gender, comorbidities and in-hospital  medications  **I**: Age, race, coexisting conditions and  medications  **J**: Sex, age, smoking, symptom, diabetes,  cardiovascular diseases, chronic liver disease, and other comorbidity  **K**: Propensity score matched  **L**: No | The dose, name and moment in time (before and during COVID-19) the medication is used is not described. The systematic review only describes the number of patients per study on ACEi/ARBs and number of patients not on an ACEi/ARBs  N of patients on patients on ACEi/ARBs  **A**: 33  **B**: 115  **C**: ACEI 1502, ARB 1394  **D**: 17  **E**: 1091  **F**: 165  **G**: 43  **H**: 188  **I**: ACEI 770, ARB 556  **J**: 103  **K**: ACEI 116, ARB 98  **L**: 42  *Measurement of*  *ACEI/ARB use*  **A**: Medical record  review  **B**: Medical record  review  **C**: Databases of health  care use  **D**: Medical record  review  **E**: Pharmacy fill records  **F**: Medical record  review  **G**: Medical record  review  **H**: Medical record  review  **I**: Medical record  review  **J**: Medical record  review  **K**: Electronic medical  records  **L**: Medical record  review | For all studies the control group is defined as COVID-19 patients not on an ACEi/ARBs.  *No. of patients not*  *on an ACEI/ARB*  **A**: 80  **B**: 247  **C**: NA  **D**: 25  **E**: 986  **F**: 136  **G**: 83  **H**: 940  **I**: non-ACEI 8140,  non-ARB 8354  **J**: 173  **K**: non-ACEI 1619, non-ARB 1637  **L**: 503 | End-point of follow-up:  **A-L**: Not available  *Study period*  **A:** Jan 1 to Feb 15 2020  **B**: Jan 15 to Mar 15 2020  **C**: Feb 21 to Mar 11 2020  **D**: Jan 11 to Feb 23 2020  **E**: Mar 1 to Apr 15 2020  **F**: Feb 1 to Apr 4 2020  **G**: Jan 5 to Feb 22 2020  **H**: Dec 31 2019 to Feb 20 2020  **I**: Dec 20 2019 to Mar 12  2020  **J**: Jan 17 to Feb 19 2020  **K**: Mar 8 to Apr 12 2020  **L**: Jan 26 to Feb 5 2020  For how many participants were no complete outcome data available?  (intervention/control)  **A-L**: NA | *1. Mortality*  No definition for mortality provided.  **A-L:** NA  Meta-analysis (**B, D, F, G, H, I, J, K** )  Pooled OR = 0.73 [95 % CI 0.5–1.07] P = 0.11  Random effect analysis  The risk of mortality  in ACEI/ARB-exposed was similar to non-ACEI/ARB exposed COVID-19 patients  Heterogenitiy (I^2^): 70.7%  P = 0.001  Sub analysis ACEI/ARB exposure and risk of mortality in COVID-19 patients with antihypertensive indication  Pooled ES 0.62 [95%CI 0.38-1.02] P=0.059  Heterogenitiy (I^2^): 74.8%  *2. IC-admission*  Not reported  *3. Hospital admission*  Not reported  *4. Length of stay*  Not reported  *5. Ventilation*  Not reported  *6. Thromboembolic complications*  Not reported | The authors conclude that that ACEI/ARB use did not increase mortality risk among patients with COVID-19. However, patient exposure to ACEI/ARBs for the treatment of hypertension was associated with a lower risk of mortality.  *Personal remarks:*  *Sensitivity analyses*  Adjusted vs unadjusted estimates  no significant increase in the mortality risk of patients with ACEI/ARB exposure regardless of unadjusted OR = 0.66 [95 % CI, 0.38–1.12] P = 0.121 or adjusted estimates OR = 0.91 [95 % CI, 0.51–1.61] P = 0.87  Studies grouped by study location  There was a significantly lower mortality risk in studies from China OR=0.65 [95 % CI 0.46−0.91] P = 0.013. There were no significant increase in mortality risk in studies form other countries (OR = 0.88, 95 % CI, 0.48–1.62, P = 0.689).  Analysis limited to studies that only included patients on ACEi/ARBs for antihypertensive indications A lower risk of mortality was observed among those who used ACEI/ARB  OR = 0.62 [95 % CI 0.38–1.02] P = 0.059 Heterogenity (I^2^): 74.85% P=0.001  After excluding studies that enrolled patients with hypertension not on antihypertensive  treatment, a meta-analysis of four studies found that ACEI/ARB exposure was associated with a lower risk of mortality compared to those on non-ACEI/ARB antihypertensive drugs  (OR = 0.48, 95 % CI, 0.29−0.81, P = 0.006; I^2^ = 0%).  *Heterogeneity:*  The I^2^ of 70.9% may represent substantial heterogeneity. The subgroup analysis described above does not explain the heterogeneity (unexplained). The heterogeneity may be explained by differences in the study populations (indication for ACEi/ARBs use, characteristics and comorbidities of the non-users, country), follow-up duration and completion of follow-up. |
| Mackay, 2020 | SR of observational studies  *Literature search MEDLINE (Ovid) and Cochrane Database of Systematic Reviews from 2003 to 4 May 2020, the World Health Organization database of COVID-19 publications and medRxiv.org through 17*  *April 2020; and ClinicalTrials.gov to 24 April 2020*  **A**: Bean, 2020 (not peer reviewed)  **B**: Feng, 2020  **C**: Li, 2020  **D**: Liu, 2020  **E**: Mancia, 2020  **F**: Mehra, 2020  **G**: Meng, 2020  **H**: Rentsch, 2020 (not peer reviewed)  **I**: Reynolds, 2020  **J**: Rossi, 2020  **K**: Yang, 2020  **L**: Peng, 2020  **M:** Zeng, 2020  **N**: Zhang, 2020  *Setting and country*  **A:** adults with COVID-19 admitted to 2  hospitals; United  Kingdom  **B:** adults with COVID-19 admitted to 3  hospitals; China  **C:** adults  with COVID-19 and HTN  admitted to 1 hospital;  China  **D:** adults with COVID-19  aged >65 y with  preexisting HTN  admitted to 3 hospitals;  China  **E:** Patients with COVID-19  aged >40 y; Lombardy,  Italy  **F:** patients  with COVID-19 admitted  to 169 hospitals in Asia,  Europe, and North  America with discharge  status available in  registry  **G:** adults  with COVID-19 and pre-existing HTN  receiving medication  and admitted to 1  hospital; China  **H:** adults  born 1945–1965 with positive COVID-19 test  result; U.S. Veterans  Health Administration  **I:** patients  with HTN and positive  COVID-19 test result in 1  health system; United  States  **J:** patients  with COVID-19; Reggio  Emilia, Italy  **K:** adults with  preexisting HTN at 1 hospital; Hubei, China  L: adults  with COVID-19 and preexisting CVD at 1 hospital; China  **M:** adults with  COVID-19 admitted to 1  hospital; China  **N:** adults  aged 18–74 y with  COVID-19 admitted to 9  hospitals; China  Source of funding and conflicts of interest:  Authors did not receive funding for this study outside of salary support. No conflicts of interest were declared. | Inclusion criteria SR: observational studies of adults in any setting examining associations  between use of ACEIs or ARBs and risks for acquiring  SARS-CoV-2 and COVID-19, SARS, or MERS; observational  studies of adults with COVID-19, SARS, or MERS,  in any setting, examining associations between ACEI or  ARB use and risks for a broad range of clinical outcomes,  including death, severity of illness (mechanical  ventilation, intensive care unit [ICU] admission, length  of stay, need for non-invasive ventilation, hospitalization,  organ dysfunction), cardiovascular events, and radiologic findings; and trials in adults with COVID-19, in  any setting, comparing laboratory or clinical outcomes between patients treated with either ACEIs or ARBs and those receiving “usual care,” placebo, or other treatments.  Exclusion criteria SR:  case reports and case series with fewer than 10 patients  *19 studies included of which 6 report on mortality, 2 on hospitalization and 1 on IC-admission*  Important patient characteristics at baseline:  **A**: n = 205  Mean age: 63 y  Male: 52%  HTN: 51%  Diabetes: 30%  Heart disease: 15%  **B**: n = 476  Median age: 53 y  Male: 57%  HTN: 24%  Diabetes: 10%  Heart disease: 8%  **C**: n = 362  Mean age: 66 y  Male: 52%  HTN: 100%  Diabetes: 35%  Heart disease: 17%  **D**: n = 46  Age, sex, and comorbid  conditions NR  **E**: n = 6272  Mean age: 68 y  Male: 63%  HTN (receiving  medication): 58%  CVD: 30%  **F**: n = 8910  Mean age: 49 y  HTN: 26%  Coronary artery  disease: 11%  Diabetes: 14%  **G**: n = 42 Median age: 65 y  Male: 57% HTN:  100%  **H**: n = 585  Median age: 66 y  Male: 95%  HTN: 72%  Diabetes: 44%  Vascular disease: 28%  **I**: n = 2573  (Demographics  reported for patients  with HTN tested for  COVID-19)  Median age: 64 y  Male: 51%  HTN: 100%  Diabetes: 40%  History of MI: 11%  CKD: 25%  **J**: n = 2653  Mean age: 63 y  Male: 50%  HTN: 18%  Diabetes: 12%  Heart failure: 6%  **K**: n = 126  Median age: 66 y  Male: 49%  HTN: 100%  Diabetes: 30%  Heart disease: 18%  **L**: n = 112  Patients with preexisting  CVD  Mean age: 62 y  Male: 47%  HTN: 82%  Diabetes: 21%  **M:** n = 75  Patients with COVID  pneumonia and HTN  Mean age: 67 y  Male: 55%  HTN: 100%  Diabetes: 31%  **N**: n = 1128  Mean age: 64 y  Male: 53%  HTN: 100%  Groups comparable at baseline?  This information is not available | The dose, name and moment in time (before and during COVID-19) the medication is used is not described  N of patients on patients on ACEi/ARBs  NA (the SR describes the N of patients receiving  ACEI or ARB with  severe Illness (%), and  non-severe illness (%)  **A**: ACEI only: 9/53 (17);  37/152 (24)  **B**: 2/124 (2); 29/352 (9)  **C**: 57/173 (32.9);  58/189 (30.7)  **D**: 4/28 (14.3); 8/18  (44.4)  **E**: NA  **F**: ACEI: 16/515 (3.1);  754/8395 (9.0)  ARB: 38/515 (7.4);  518/8395 (6.2)  **G**: 4/17 (23.5);  12/25 (48)  **H**: Hospitalization:  147/297 (49.5)  ICU admission:  69/122 (56.6)  Not hospitalized:  108/288 (37.5)  **I**: NA  **J**: 501/1075 (46.6);  317/1578 (20.1  **K**: 15/50 (30.0); 28/76  (36.8)  **L**: 3/16 (18.6); 19/96  (19.8)  **M:** 15/30 (50);  13/45 (29)  **N**: NA | For all studies the control group is defined as COVID-19 patients not on an ACEi/ARBs. No further details are available.  *No. of patients not*  *on an ACEI/ARB*  **A-N:** NA | Study period:  **A**: 3/1/20–3/22/20  **B**: 1/1/20–2/15/20  **C**: /15/20–3/15/20  **D**: Time varied by site (range, 12/27/19–2/29/20);  **E**: NA  **F**: 12/20/19–3/15/20;  **G**: 1/11/20–2/23/20  **H**: 2/8/20–3/30/20  **I**: 3/1/20–4/15/20  **J**: 2/27/20–4/2/20  **K**: 1/5/20–2/22/20  **L**: 1/20/20–2/15/20  **M:** 1/5/20–3/8/20  **N**: 12/31/19–2/20/20  For how many participants were no complete outcome data available?  (intervention/control)  **A-N**: NA | *1. Mortality*  No definition for mortality provided.  Unadjusted  **A:** (Mortality and transfer  to critical care  within 7 d of  symptom onset)  ACEi OR 0.64 (0.28–1.43)  ACEi aOR 0.29 (0.10–0.75)  **C:** OR 0.76 [95%CI 0.43–1.33]  **E:** Assisted ventilation or  Death ACEI:aOR 0.91 (0.69–1.21)  ARB: aOR 0.83  (0.63–1.10)  **F**: ACEI OR 0.33 [95% CI 0.19–0.54]  ARB OR 1.21 [95%CI 0.86–1.71]  I: severe covid: ICU admission, use of  noninvasive or  mechanical  ventilation, or  death  Overall  ACEi (mean diff for severe covid) −1.9 (−6.6 to 2.8)  ARB (mean diff) −1.4 (−6.1 to 3.3)  ACEi or ARB mean diff −0.1 (−3.7 to 3.5)  Thiazide diuretic (mean diff) −3.4 (−8.3 to 1.6)  hypertensive patients  ACE inhibitor (mean diff for severe covid) −3.3 (−8.2 to 1.7)  ARB mean diff 0.1 (−4.8 to 4.9)  ACE inhibitor or ARB mean diff −0.5 (−4.3 to 3.2)  Thiazide diuretic (mean diff)  0.6 (−4.5 to 5.7)  **J**: HR for death with ACEI  0.8 [95%CI 0.50–1.3]  **K**: OR 0.32 [95%CI 0.07–1.51]  **M:** OR 0.65 [95%CI 0.12–3.58]  **F**: Adjusted for severe illness  ACEI OR 0.33 [95%CI 0.20–0.54]  ARB OR 1.23 [95% CI 0.87–1.74]  **N:** Adjusted HR (age, sex, comorbid conditions, and in-hospital medications) 0.42 [95%CI 0.19–0.92]  *2. IC-admission*  A: see mortality  H: unadjusted OR 1.94 [95%CI 1.30–2.90] adjusted OR 1.69  [95%CI 1.01–2.84]  I: see mortality  *3. Hospital admission*  **H:** unadjusted OR 1.63 [95%CI 1.17–2.27] Adjusted (age, race, comorbid conditions, and Veterans Aging Cohort Study index) aOR 1.24  [95%CI 0.79–1.95]  **J**: aHR with ACEI (age, sex, and Charlson comorbidity score): 1.13 [95%CI 1.1–1.5] aHR (age, sex, and Charlson comorbidity score, and restricted to patients with CVD) with ACEI  aHR 1.12 [95%CI 0.82–1.54]  *4. Length of stay*  Not reported  *5. Ventilation*  E: see mortality  I: see mortality  *6. Thromboembolic complications*  Not reported | The authors conclude that no indication exists to prophylactically  stop ACEI or ARB treatment because of concerns  about COVID-19. |

1= Reported for hypertensive patients; 2= Calculated for 610 COVID 19 patients out of total of 49 277; 3 = Patients tested for COVID-19; 4= Patients aged over 35 years suspected of or diagnosed with COVID-19; 5= Not on any antihypertensive drug; 6= Before matching; 7= After matching; 8= Other regimens; 9 = Reported for COVID-19-positive patients (187 out of 288 suspected of or diagnosed patients)
