# Supplementary material for: The effects of ACE2 expression mediating pharmacotherapy in COVID-19 patients
Source: Neth Heart J. 2021 Apr 16;29(Suppl 1):20–34. doi: 10.1007/s12471-021-01573-8 (PMC8050813; doi:10.1007/s12471-021-01573-8)
Supplement: Supplementary file 4 — Table S4 Evidence table for intervention studies (randomised controlled trials and non-randomised observational studies [cohort studies, case-control studies, case series]) [file 12471_2021_1573_MOESM4_ESM.docx]

**Table S4** Evidence table for intervention studies (randomised controlled trials and non-randomised *observational* studies [cohort studies, case-control studies, case series])^1^

*This table is also suitable for diagnostic studies (screening studies) that compare the effectiveness of two or more tests. This only applies if the test is included as part of a test-and-treat strategy – otherwise the evidence table for studies of diagnostic test accuracy should be used.*

| **Study reference** | **Study characteristics** | **Patient characteristics ^2^** | **Intervention (I)** | **Comparison / control (C) ^3^** | **Follow-up** | **Outcome measures and effect size ^4^** | **Comments** |
| --- | --- | --- | --- | --- | --- | --- | --- |
| Felice, (2020) | Type of study: retrospective observational study  Setting and country: hypertensive patients who presented to the emergency department, Italy  Funding and conflicts of interest: Funding not reported. No conflicts of interest declared. | Inclusion criteria: all consecutive hypertensive subjects who presented to the emergency department (ED) with acute respiratory symptoms/fever, and were diagnosed with COVID-19 infection  Exclusion criteria: NA  N total at baseline: 133  ACEI N=40  ARB N=42  Not on RAAS inhibitors N=51  Important prognostic factors^2^:  *Mean age (SD):*  *ACEI: 73.1 (11.5)*  *ARB: 69.0 (13.4)*  *Not on RAAS: 76.2 (11.9)*  *Sex male (%):*  *ACEI: 28 (70)*  *ARB: 31 (74)*  *Not on RAAS 27 (53)*  Groups comparable at baseline? No significant differences were observed for all demographics and clinical parameters, except for the history of chronic heart failure, which was more frequently observed in hypertensive patients not on RAAS inhibitors (31%; P=0.007). | Hypertensive patients on ACEI  Patients on ACEI were chronically using ACEIs. 70% were taking ramipril.  Hypertensive patients on ARB  Patients on ARB were chronically using ARBs. Olmesartan was used in more than 50% of patients. | Hypertensive patients using other blood pressure lowering medications then ACEI or ARBs. | Length of follow-up:  Mean 15.8 ± 8.6 days  Loss-to-follow-up:  NA  Incomplete outcome data:  No | *1. Mortality*  OR of 0.41 (CI 95%, 0.18-0.92; P=0.030)  aOR (gender, BMI, days with symptoms prior to admission, previous cardiovascular events, diabetes and cancer) OR 0.56, CI95% 0.17-1.83, P=0.341  *2. IC-admission*  *(semi intensive care/ic)*  OR 0.36 [95%CI 0.17-0.75] P=0.007  aOR (gender, BMI, days with symptoms prior to admission, previous cardiovascular events, diabetes and cancer) OR 0.25 [CI95% 0.09-0.66] P=0.006  Admission to semi-intensive/intensive care units, less likely to occur in hypertensive patients using ARB or ACEI (significant).  *3. Hospital admission*  OR 0.45 [95%CI 0.09-2.24]  P=0.327  aOR 0.39 [95%CI 0.05-2.94] P=0.365  *4. Length of stay*  Not reported  *5. Ventilation*  Defined as Oxygen therapy  OR 0.46 [95%CI 0.18-1.18] P=0.107 aOR 0.51 [95%CI 0.15-1.78] P=0.292  Defined as non-invasive ventilation  OR 0.70 [95%CI 0.34-1.44] P=0.336  aOR 0.58[95%CI 0.21-1.60] P=0.296*6.*  *6. Thromboembolic complications*  Not reported |  |
| Gao (2020) | Type of study: retrospective observational  Setting and country: Covid patients admitted to the hospital, China (for our outcome measures only hypertensive patients)  Funding and conflicts of interest: Not reported and no conflicts of interest declared. | Inclusion criteria: All patients admitted  to Huo Shen Shan Hospital, Wuhan, China, from 5 February to 15 March 2020, with confirmed COVID-19  Exclusion criteria: NA  Patients were confirmed or suspected COVID-19 patients  N total at baseline:  N total: 2877  N hypertensive patients on antihypertensive medication=710 (183 on RAAS inhibiter, 527 on non-RAAS inhibitor)  Important prognostic factors^2^:  *age ± SD:*  *Non-RAAS inhibitor: 64.84 ± 11.19*  *RAAS inhibitor: 62.64 ± 11)*  *Sex (%male):*  *Non-RAAS inhibitor: 266 (50.5%)*  *RAAS inhibitor: 104 (56.8%)*  Groups comparable at baseline?  Yes, for all (symptoms at admission, blood pressure, medical history) except shivering | Hypertensive patients on ACEI and/or ARBs | Hypertensive patients not on RAAS (but on B blockers, antidiuretics etc) | Length of follow-up: median 21 (12–  32) days  Loss-to-follow-up:  NA  Incomplete outcome data:  NA | *1. Mortality*  RAASI: 4/183 (2.2%)  Non-RAASI: 19/527 (3.6%)  OR  Unadjusted 0.60 (95% CI 0.20–1.76) P= 0.354  Adjusted (age, sex, medical history of diabetes, insulin-treated diabetes, myocardial infarction, underwent PCI/CABG, renal failure, stroke, heart failure, and COPD)  ORa 0.85 [95% CI 0.28–2.58] P= 0.774  Propensity score adjusted 0.93 [95% CI 0.31–2.84] P=0.901  *2. IC-admission*  Not reported  *3. Hospital admission*  Not reported  *4. Length of stay*  Not reported  *5. Ventilation*  Defined as invasive mechanical ventilation  RAASI: 5 (2.7%)  Non-RAASI: 25 (4.7%) P=0.292  There is no significant difference in mechanical ventilation between hypertensive patients on RAAS inhibitors and on non-Raas inhibitors.  *6. Thromboembolic complications*  Not reported |  |
| Imam (2020) | Type of study: Retrospective, multicenter cohort  Setting and country: patients hospitalized with COVID-19, US  Funding and conflicts of interest: none and none declared | Inclusion criteria: patients hospitalized with SARS-CoV-2 infection demonstrated by a positive RT-PCR on nasopharyngeal swab per world health organization (WHO) guidance between March 1-April 1,2020  Exclusion criteria: NA  N total at baseline:  Total N=1305  ACEI or ARBs N=565 (43.3%)  NSAIDS N= 466 (35.7%)  Important prognostic factors^2^:  Not available per group  *age ± SD: 61.0 ±16.3*  *Male Sex 702 (53.8%)*  Groups comparable at baseline? NA because the groups are not defined as in the PICO | NSAID use  ACEI/ARBs use  No further details available | patients hospitalized with SARS-CoV-2 | Length of follow-up: NA  Loss-to-follow-up:  NA  Incomplete outcome data:  NA | *1. Mortality*  Univariate analysis  NSAIDS use  OR 0.55 [95% CI0.39-0.78] P=.001  ACE-I/ARB use OR 1.55 [95% CI 1.15-2.10] P=.004  Multivariate analysis (Age, Initial Serum Creatinine, CCI, NSAID, HTN, ACE-I/ARB use, CKD)  NSAID use  OR 0.57 [95%CI 0.40-0.82] P=0.002  ACE-I/ARB use  OR 1.20 [95%CI 0.86-1.68] P=0.278  *2. IC-admission*  Not reported  *3. Hospital admission*  Not reported  *4. Length of stay*  Not reported  *5. Ventilation*  Not reported  *6. Thromboembolic complications*  Not reported |  |
| Jung (2020) | Type of study: population based cohort using a database of de-identified COVID-19 patient data  Setting and country: patients admitted to the hospital and patients not admitted to the hospital, Korea  Funding and conflicts of interest: No funding and no conflicts of interest declared. | Inclusion criteria: patients with COVID-19 who were ≥18 years old  Exclusion criteria: NA  N total at baseline:  N total = 5179  N hospitalized (with clinical outcomes) = 1954  N (hospitalized RAASi users) = 377  N (hospitalized non RAASi users) = 1577  Important prognostic factors for total:  *age ± SD:*  *RAASi:62.5 ± 14.7*  *Non-RAASI: 41.5 ± 16.6*  *Sex (% male):*  *RAASi: 400 (52)*  Non-RAASi: *1895 (43)*  Charlson comorbidity index (mean (SD))  RAASi: 3.3 (2.8)  Non-RAASi: 1.2 (1.9)  Groups comparable at baseline?  No, there is a significant difference between the groups on all included factors (age, sex, comorbidities, Charlson comorbidity index, immunosuppression) | RAAS inhibitor users were defined as patients with RAAS inhibitor use at 1–30 days before the index date | Patients who had never received RAAS inhibitors or had received them at 31–365 days before the index date. A prescription duration of ≥7 days was required to define drug use. | Length of follow-up:  All patients were followed until the first instance of death or April 8, 2020  Loss-to-follow-up:  NA  Incomplete outcome data:  NA | *1. Mortality*  Defined as in-hospital mortality  Observed for 33/377 RAAS inhibitor users (9%) and for 51/1577 nonusers (3%) (p<0.001)  Univariate analysis (total group)  OR 3.88 [95%CI 2.48 6.05] P<0.001  Multivariate analysis (total group, adjusted for age, sex, Charlson Comorbidity Index, immunosuppression, and hospital type))  adjusted OR, 0.88 [95% CI 0.53–1.44] p=0.60  RAAS inhibitor use was not independently associated with a higher risk of mortality among COVID-19 patients  Multivariate analysis (adjusted for age, sex, Charlson Comorbidity Index, immunosuppression, and hospital type) hypertensive patients  adjusted OR 0.71 [95% CI 0.40–1.26] p=0.25  RAAS inhibitor use was not independently associated with a higher risk of mortality among hypertensive COVID-19 patients.  *2. IC-admission*  Not reported  *3. Hospital admission*  Not reported  *4. Length of stay*  Not reported  *5. Ventilation*  Defined as mechanical ventilation)  OR 3.74 [95% CI 1.91 7.34] P<0.001  Adjusted OR 1.03 [0.50 2.13] P=0.93  RAAS inhibitor use was not independently associated with a higher risk of mechanical ventilation  *6. Thromboembolic complications*  Defined as acute cardiac event  OR 1.69 [95% CI 1.19 2.39] P= 0.003  Adjusted OR 0.88 [95% CI 0.59 1.31] P=0.53  RAAS inhibitor use was not independently associated with a higher risk of an acute cardiac event |  |
| López-Otero (2020) | Type of study: Single-center, retrospective, observational cohort study  Setting and country: Hospitalized and non-hospitalized patients, Spain  Funding and conflicts of interest: Funding information NA, no conflict of interest declared. | Inclusion criteria: all cases of laboratory-confirmed SARS-CoV-2  infection in the area,  Exclusion criteria:  NA  N total at baseline:  Total N=965  ACEI/ARB N= 213 No ACEI/ARB N = 755  Important prognostic factors^2^:  *age ± SD:*  ACEI/ARB *: 72.1 ± 13.2*  No ACEI/ARB*: 56.0 ± 20.5*  *Sex (%female):*  ACEI/ARB *: 43.8 59.5*  No ACEI/ARB*: 59.5*  Groups comparable at baseline?  The cohort of patients under ACEI/ARB was older (72.1 *±*  13.2 vs 56.0 *±*  20.5; P < .01) and had more cardiovascular risk factors (hypertension,  diabetes, smoking, and dyslipidemia) and cardiovascular comorbidities (coronary artery diseases and ventricular dysfunction) than the  cohort without ACEI/ARB. There were fewer women in the ACEI/ARB group (43.8% vs 59.5%; P < .01). Renal impairment and peripheral vasculopathy were also more prevalent in patients taking ACEI/ARB. | Use of ACEI, ARB or both  Of the COVID-19 patients, 210 (21.8%) were under ACEI or ARB treatment at the time of diagnosis; of these, 165 (78.57%) were taking them for more than 1 year. | No use of ACEI or ARB | Length of follow-up:  Study period from 10 March to 6 April  Loss-to-follow-up:  NA  Incomplete outcome data:  NA | *1. Mortality*  Univariate analysis  OR 1.49 [95%CI 0.73-3.06] p=0.276  Multivariate analysis (adjusted for fever,  oxygen saturation  < 95%, age, sex, obesity, health personnel, dependency status,  hypertension, diabetes mellitus, dyslipidemia, arterial disease, heart disease, atrial fibrillation, pneumonia, chronic renal disease, cerebrovascular disease, autoimmune disease, anticoagulation, beta-blockers)  OR 0.62 [95%CI 0.17-2.26] P=0.468  Propensity score matching ACEI/ARBs OR 0.47 [95%CI 0.14-1.64] P=0.239  Previous treatment with ACEI/ARB (combined and individually)  showed no impact on mortality.  *2. IC-admission*  Univariate analysis  OR 1.36 [95%CI 0.64-2.90] P=0.427  Multivariate analysis (adjusted for arterial oxygen saturation  < 95%, diabetes  mellitus, hypoxemia, hypercapnia, lymphocytes, creatinine, elevated troponin,  ferritin, C-reactive protein, interleukin-6)  OR 0.87 [95%CI 0.30-2.50] P=0.798  Previous treatment with ACEI/ARB (combined and individually)  showed no impact on IC admission  *3. Hospital admission*  Univariate analysis  OR 2.27 [95% CI 1.63-3.16] P<.001  Multivariate analysis (adjusted for days with symptoms, fever, arterial oxygen saturation  < 95%, age, sex,  health personnel, institutionalized, dependency status, dementia, hypertension,  dyslipidemia, ventricular dysfunction, lung disease, previous cancer, hypothyroidism,  antiplatelet therapy)  OR 0.85 [95%CI 0.45-1.64] P=0.638  Previous treatment with ACEI/ARB (combined and individually)  showed no impact on hospital admission.  *4. Length of stay*  Not reported  *5. Ventilation*  Not reported  *6. Thromboembolic complications*  Defined as heart failure  Univariate analysis  OR 2.20 [95%CI 1.09-4.44] P=0.028  Multivariate analysis (adjusted for fever,  oxygen saturation  < 95%, age, sex, obesity, health personnel, dependency status,  hypertension, diabetes mellitus, dyslipidemia, arterial disease, heart disease, atrial fibrillation, pneumonia, chronic renal disease, cerebrovascular disease, autoimmune disease, anticoagulation, beta-blockers)  OR 1.37 [95%CI 0.39-4.77] P=0.622 | Subgroup of patients requiring hospitalization,  The absence of an impact on mortality and on heart failure remained both in the multivariate analysis and in the propensity score model, including in the evaluation of treatment taken for more than 1 year |
| Selçuk (2020) | Type of study: observational  Setting and country: hypertensive patients  admitted due to Covid-19 infection, Turkey  Funding and conflicts of interest: The authors have not declared a specific grant for this research from any funding agency in the public, commercial or not-for-profit sectors. No conflicts of interest were declared. | Inclusion criteria: consecutive  hypertensive patients admitted to our centers due to Covid-  19 infection  Exclusion criteria:  Patients with the absence of in-hospital  clinical data, heart failure patients with hypertension were not included in the study  N total at baseline:  N total = 113  ACE inh/ARBs users N=74  Non-users N=39  Important prognostic factors^2^:  *age ± SD:*  ACE inh/ARBs users: 67 ± 11  Non-user: 58 ± 10  *Sex (N, % male):*  ACE inh/ARBs users: 36 (48.6)  Non-user: 23 (59.0)  Groups comparable at baseline?  The patients in the ACE inh/ARBs group were older. The frequency  of coronary artery disease was significantly higher in patients using an ACE inh/ARBs as anti-hypertensive treatment  (p = .009). The other baseline features and medical treatments  were indifferent between the groups. patients in the ACE inh/ARBs group had  significantly higher white blood cell (WBC) and neutrophils  count | Patients on ACEI and/or ARBs | All patients were on other than ACE inh/ARBs antihypertensive  therapy unless no contraindication was present. | Length of follow-up:  NA  Loss-to-follow-up:  INA  Incomplete outcome data:  NA | *1. Mortality*  Univariate analysis  OR 6.30 (95%CI 2.03–19.58)  P0.001  Multivariate analysis (adjusted for age, coronary artery disease, ACE inh/ARBs  use, D-dimer, WBC count, creatinine, plasma glucose, and lactate dehydrogenase) OR 3.66 (95%CI:  1.11–18.18) p= .032 ACEI/ARB use is an independent predictor of inhospital mortality.  Kaplan-Meir curve analysis displayed that patients on ACE inh/ ARBs therapy had higher incidence of in-hospital death than those who were not [log rank test p value <.001  *2. IC-admission*  ACE inh/ARBs users: 37 (50.0)  Non-user: 7 (17.9) ) P=.001  *3. Hospital admission*  Not reported  *4. Length of stay (days)*  ACE inh/ARBs users: 9 ± 6  Non-user: 8 ± 4  P=0.524  *5. Ventilation*  Defined as endotracheal intubation ACE inh/ARBs users: 33 (44.6)  Non-user: 4 (10.3) P<0.001*6.*  *6. Thromboembolic complications*  Not reported |  |
| Zhou (2020) | Type of study: Retrospective, single center cohort  Setting and country: discharged patients with COVID-19, China  Funding and conflicts of interest: not reported and none declared | Inclusion criteria: confirmed patients with COVID-19 at Wuhan Fourth Hospital discharged from January 25 to February 20, 2020.  Exclusion criteria: NA  N total at baseline:  Total N=110  History of hypertension N=36 (32.7%)  ACEI or ARBs N=15 (41.7%) (43.3%)  Other antihypertensive drugs (control) N=21 (58.3%)  Important prognostic factors^2^:  *Age ± SD*  ACEI/ARBs users:  58.5 ± 10.1 years  Other: 69.2 ± 7.5 years  *Male Sex: N (%)*  ACEI/ARBs users:  *9 (60%)*  Other: 10 (47.6%)  Groups comparable at baseline? Age was significant different (p=0.001) between groups. Other factors were comparable. | Patients taking ACEI/ARBs  No further details available | Patients taking other antihypertensive drugs.  No further details available | Length of follow-up: NA  Loss-to-follow-up:  NA  Incomplete outcome data:  NA | *1. Mortality*  ACEI/ARBs users: 2 (13.3%)  Non-user: 5 (23.8%) P<0.676*.*  *2. IC-admission*  Not reported  *3. Hospital admission*  Not reported  *4. Length of stay*  *Mean (SD)*  *I: 10.1(5.2)*  *C: 11.7(6.0)*  *P=0.405*  *5. Ventilation*  Not reported  *6. Thromboembolic complications*  Not reported |  |

**Notes:**

1. **Prognostic balance between treatment groups is usually guaranteed in randomized studies, but non-randomized (observational) studies require matching of patients between treatment groups (case-control studies) or multivariate adjustment for prognostic factors (confounders) (cohort studies); the evidence table should contain sufficient details on these procedures**
2. **Provide data per treatment group on the most important prognostic factors [(potential) confounders]**
3. **For case-control studies, provide sufficient detail on the procedure used to match cases and controls**
