# Supplementary material for: The effects of ACE2 expression mediating pharmacotherapy in COVID-19 patients
Source: Neth Heart J. 2021 Apr 16;29(Suppl 1):20–34. doi: 10.1007/s12471-021-01573-8 (PMC8050813; doi:10.1007/s12471-021-01573-8)
Supplement: Supplementary file 5 — Table S5 Quality assessment for systematic reviews of RCTs and observational studies Based on AMSTAR checklist (Shea et al.; 2007, BMC Methodol 7: 10; https://doi.org/10.1186/1471-2288-7-10) and PRISMA checklist (Moher et al. 2009, PLoS Med 6: e1000097; https://doi.org/10.1371/journal.pmed1000097) [file 12471_2021_1573_MOESM5_ESM.docx]

**Table S5** Quality assessment for systematic reviews of RCTs and observational studies

Based on AMSTAR checklist (Shea et al.; 2007, BMC Methodol 7: 10; doi:10.1186/1471-2288-7-10) and PRISMA checklist (Moher et al 2009, PLoS Med 6: e1000097; doi:10.1371/journal.pmed1000097)

| **Study**  **First author, year** | **Appropriate and clearly focused question?^1^**  **Yes/no/unclear** | **Comprehensive and systematic literature search?^2^**  **Yes/no/unclear** | **Description of included and excluded studies?^3^**  **Yes/no/unclear** | **Description of relevant characteristics of included studies?^4^**  **Yes/no/unclear** | **Appropriate adjustment for potential confounders in observational studies?^5^**  **Yes/no/unclear/not applicable** | **Assessment of scientific quality of included studies?^6^**  **Yes/no/unclear** | **Enough similarities between studies to make combining them reasonable?^7^**  **Yes/no/unclear** | **Potential risk of publication bias taken into account?^8^**  **Yes/no/unclear** | **Potential conflicts of interest reported?^9^**  **Yes/no/unclear** |
| --- | --- | --- | --- | --- | --- | --- | --- | --- | --- |
| Zhang, 2020 | Yes | Yes | Yes | No (number of events not reported per study) | Unclear (some studies in the SR adjusted for confounders but not all) | Yes | Yes (subgroup analysis were performed to make reasonable combining of studies) | Yes (publication bias could not be assessed because less than 10 studies were included in the meta-analysis) | No |
| Mackay, 2020 | Yes | Yes | No (no exclusion reason were provided) | Yes | Unclear (some studies in the SR adjusted for confounders but not all) | Yes | Yes (a meta-analysis might not be appropriate and was not performed) | No | No (not for included studies) |

1. **Research question (PICO) and inclusion criteria should be appropriate and predefined**
2. **Search period and strategy should be described; at least Medline searched; for pharmacological questions at least Medline + EMBASE searched**
3. **Potentially relevant studies that are excluded at final selection (after reading the full text) should be referenced with reasons**
4. **Characteristics of individual studies relevant to research question (PICO), including potential confounders, should be reported**
5. **Results should be adequately controlled for potential confounders by multivariate analysis (not applicable for RCTs)**
6. **Quality of individual studies should be assessed using a quality scoring tool or checklist (Jadad score, Newcastle-Ottawa scale, risk of bias table etc.)**
7. **Clinical and statistical heterogeneity should be assessed; clinical: enough similarities in patient characteristics, intervention and definition of outcome measure to allow pooling? For pooled data: assessment of statistical heterogeneity using appropriate statistical tests (e.g. Chi-square, I^2^)?**
8. **An assessment of publication bias should include a combination of graphical aids (e.g., funnel plot, other available tests) and/or statistical tests (e.g., Egger regression test, Hedges-Olken). Note: If no test values or funnel plot included, score “no”. Score “yes” if mentions that publication bias could not be assessed because there were fewer than 10 included studies.**
9. **Sources of support (including commercial co-authorship) should be reported in both the systematic review and the included studies. Note: To get a “yes,” source of funding or support must be indicated for the systematic review AND for each of the included studies.**
