# Supplementary material for: The effects of ACE2 expression mediating pharmacotherapy in COVID-19 patients
Source: Neth Heart J. 2021 Apr 16;29(Suppl 1):20–34. doi: 10.1007/s12471-021-01573-8 (PMC8050813; doi:10.1007/s12471-021-01573-8)
Supplement: Supplementary file 6 — Table S6 Risk of bias for intervention studies (observational: non-randomised clinical trials, cohort and case-control studies) [file 12471_2021_1573_MOESM6_ESM.docx]

**Table S6** Risk of bias table for intervention studies (observational: non-randomised clinical trials, cohort and case-control studies)

| **Study reference**  (first author, year of publication) | **Bias due to a non-representative or ill-defined sample of patients?**^1^  (unlikely/likely/unclear) | **Bias due to insufficiently long, or incomplete follow-up, or differences in follow-up between treatment groups?^2^**  (unlikely/likely/unclear) | **Bias due to ill-defined or inadequately measured outcome ?^3^**  (unlikely/likely/unclear) | **Bias due to inadequate adjustment for all important prognostic factors?^4^**  (unlikely/likely/unclear) |
| --- | --- | --- | --- | --- |
| Felice, (2020) | Unlikely (hypertensive confirmed covid patients) | unlikely | Mortality: Unlikely  IC-admission: unclear (admitted to semi/ intensive care)  Hospital admission: unclear (criteria not defined)  Oxygen therapy: unlikely  Non-invasive ventilation: unlikely | Mortality: Unlikely (adjusted for for gender, BMI, days with symptoms prior to admission, previous cardiovascular events, diabetes and cancer)  IC admission: unlikely (adjusted for gender, BMI, days with symptoms prior to admission, previous cardiovascular events, diabetes and cancer)  Hospital admission: unlikely (adjusted) *Oxygen therapy:* unlikely (adjusted)  Non-invasive ventilation: unlikely (adjusted) |
| Gao (2020) | Unlikely (hypertensive confirmed or suspected covid patients) | Unlikely | Mortality: unlikely  Invasive mechanical ventilation: unlikely | Mortality: Unlikely (age, sex, medical history of diabetes, insulin-treated diabetes, myocardial infarction, underwent PCI/CABG, renal failure, stroke, heart failure, and COPD)  Invasive mechanical ventilation: likely (no correction for confounders) |
| Imam (2020) | Unclear (the groups are different then in the PICO so samples not possible to assess) | Unclear (information is missing on how many patients were still hospitalized at the moment of analysis) | Mortality: unlikely | Unlikely (multivariate analysis performed) |
| Jung (2020) | unlikely | Unclear (information is missing on how many patients were still hospitalized at the moment of analysis) | Mortality: unlikely  Mechanical ventilation: unlikely  Acute cardiac event: unlikely | Mortality: Unlikely (age, sex, Charlson Comorbidity Index, immunosuppression, and hospital type)  Mechanical ventilation: unlikely (age, sex, Charlson Comorbidity Index, immunosuppression, and hospital type)  Acute cardiac event: unlikely (adjusted for age, sex, Charlson Comorbidity Index, immunosuppression, and hospital type) |
| López-Otero (2020) | Unlikely (confirmed COVID patients) | Unclear (unclear how long the follow up duration was and how many patients are still hospitalized) | Mortality: unlikely  IC admission: unclear  Heart failure: according to the European Society of Cardiology guidelines | Unlikely (adjusted for days with symptoms, fever, arterial oxygen saturation  < 95%, age, sex, health personnel, institutionalized, dependency status, dementia, hypertension, dyslipidemia, ventricular dysfunction, lung disease, previous cancer, hypothyroidism,  antiplatelet therapy)  Hospital admission: unlikely  IC-admission: unlikely  Heart failure: unlikely |
| Selçuk (2020) | Unlikely (hypertensive confirmed covid patients) | Unclear (there is no information available on follow-up duration) | Mortality: unlikely  IC admission: unclear (criteria not described, may depend on capacity)  Length of stay: unclear (discharge criteria not described, may depend on capacity)  Ventilation: unlikely | Mortality: unlikely (adjusted for adjusted for age, coronary artery disease, ACE inh/ARBs use, D-dimer, WBC count, creatinine, plasma glucose, and lactate dehydrogenase)  IC-admission: likely (no correction for confounders)  Length of stay: likely (no correction for confounders)  Ventilation: likely (no correction for confounders) |
| Zhou (2020) | Unlikely | Unlikely (all included patients have been discharged) | Mortality: unlikely  Length of stay: unclear (discharge criteria not described, may depend on capacity) | Mortality: likely (no correction for confounders)  Length of stay: likely (no correction for confounders) |

1. **Failure to develop and apply appropriate eligibility criteria: a) case-control study: under- or over-matching in case-control studies; b) cohort study: selection of exposed and unexposed from different populations.**
2. **2 Bias is likely if: the percentage of patients lost to follow-up is large; or differs between treatment groups; or the reasons for loss to follow-up differ between treatment groups; or length of follow-up differs between treatment groups or is too short. The risk of bias is unclear if: the number of patients lost to follow-up; or the reasons why, are not reported.**
3. **Flawed measurement, or differences in measurement of outcome in treatment and control group; bias may also result from a lack of blinding of those assessing outcomes (detection or information bias). If a study has hard (objective) outcome measures, like death, blinding of outcome assessment is not necessary. If a study has “soft” (subjective) outcome measures, like the assessment of an X-ray, blinding of outcome assessment is necessary.**
4. **Failure to adequately measure all known prognostic factors and/or failure to adequately adjust for these factors in multivariate statistical analysis.**
